# Supplementary material for: Extended pharmacodynamic responses observed upon PROTAC-mediated degradation of RIPK2
Source: Commun Biol. 2020 Mar 20;3:140. doi: 10.1038/s42003-020-0868-6 (PMC7083851; doi:10.1038/s42003-020-0868-6)
Supplement: Supplementary file 1 — Supplementary Information [file 42003_2020_868_MOESM1_ESM.pdf]

Supplementary Information for Extended pharmacodynamic responses observed upon PROTAC-mediated degradation of RIPK2

Cell Name	Cell type	Average half-life (uncorrected mean) hours
B	Primary	275.4
HepG2	Cell line	26.9
Monocytes	Primary	49.1
NK	Primary	500
THP1	Cell line	51.8
T	Primary	254

Supplementary Figure 1. Determination of RIPK2 half-live in cells. Protein half-lives were computed according to methods described by Schwanhausser[1]. SILAC enables the determination of protein turnover by measuring the ratio of protein labelled with heavy amino acids and light amino acids at different time points[2].

a)

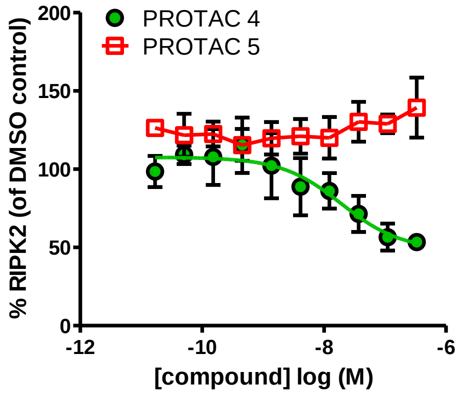

b)

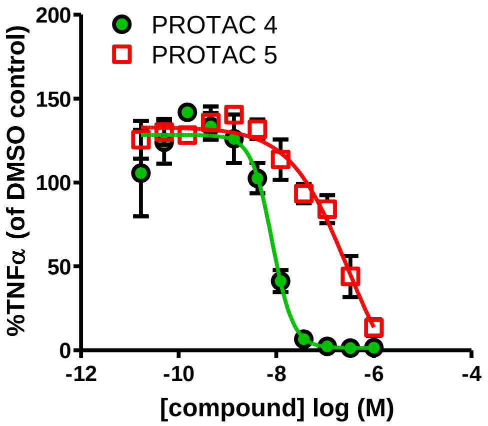

c)

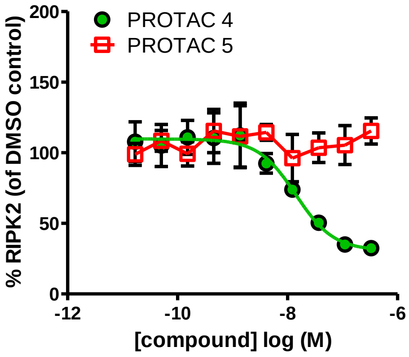

Supplementary Figure 2. *In vitro* comparison of PROTAC **4** and control PROTAC **5** in human PBMCs following 6 h incubation showing **a)** RIPK2 levels and **b)** MDP-induced TNFα inhibition. **c)** *In vitro* comparison of RIPK2 degradation by PROTAC **4** and control PROTAC **5** in human PBMCs following 24 h incubation (n=3).

a)

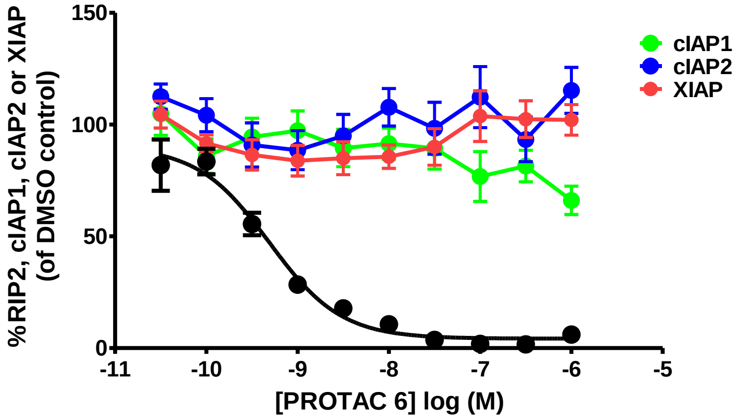

b)

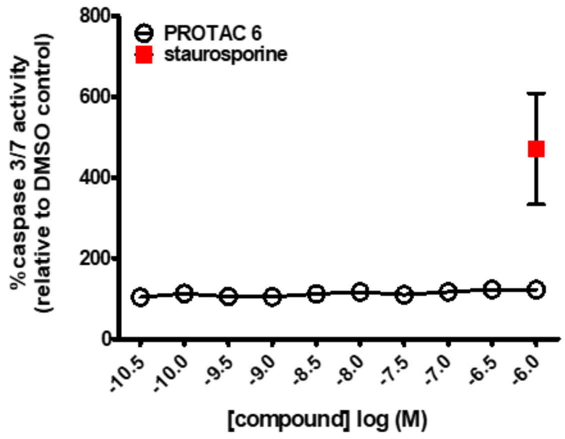

c)

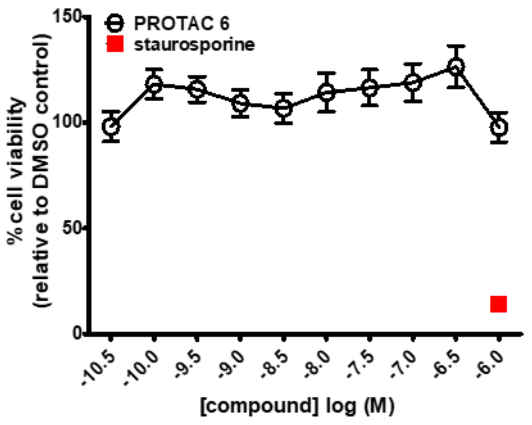

Supplementary Figure 3. a) The effect of PROTAC **6** on RIPK2 levels and IAP family members in human PBMCs following 24 h incubation. While PROTAC **6** potently degrades RIPK2, there is no change in protein levels of cIAP2 or XIAP as measured by capillary immunoassay up to concentrations of 1uM. At 1uM there is ~25% reduction in cIAP1 levels. The effect of PROTAC **6** on **b)** caspase activation and **c)** on cell viability in human PBMCs. PBMCs were treated with PROTAC **6** for 24h**.** Cell viability was assessed by using Cell Titer Glo Kit (Promega) while Caspases 3 and 7 activities were detected by using Capase-Glo 3/7 Kit (Promega) according to the manufacturer's instructions. Samples were analysed using the EnVision Multiplate Reader and associated software (Perkin EnVision Manager version 1.13.30009.1401). The intensity of luminescence obtained for the wells containing no cells (background) was subtracted from the intensity obtained for each sample. There was no effect on caspases 3/7 activation or on cell viability within tested range of compound concentration. Staurosporine (1 µM) was used as positive control for caspase activation that leads to reduction in cell viability.

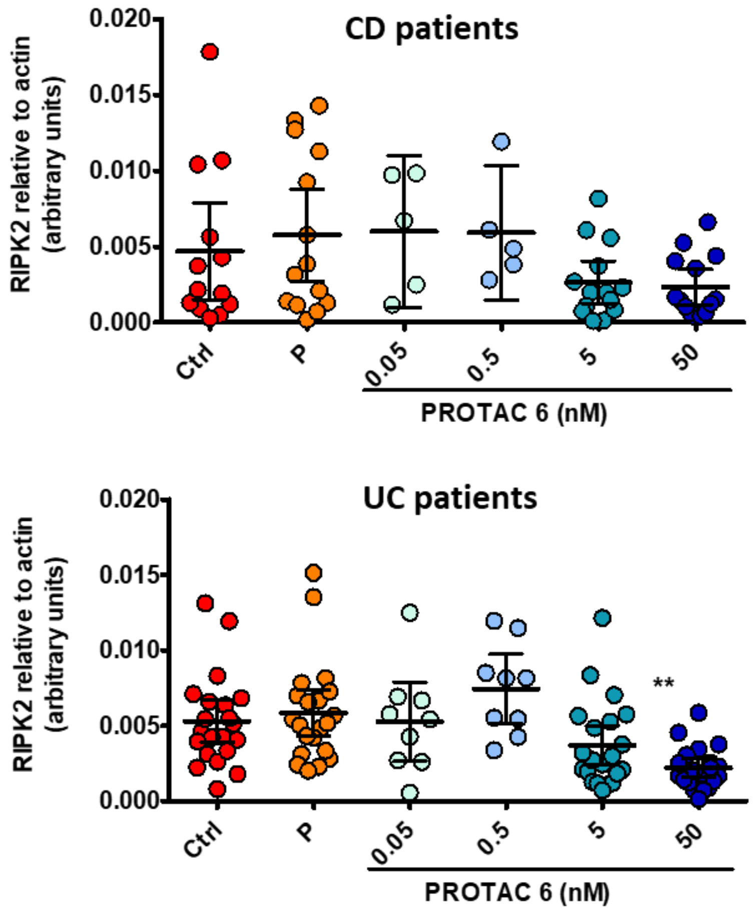

Supplementary Figure 4. RIPK2 levels from human biopsy samples shown in Figure 4e. RIPK2 quantification via Western blot for DMSO control groups (Ctrl), 1µM Prednisolone groups (P) and PROTAC 6 treated groups. P values calculated by ANOVA Dunett test comparing control vs. PROTAC (** P<0.01).

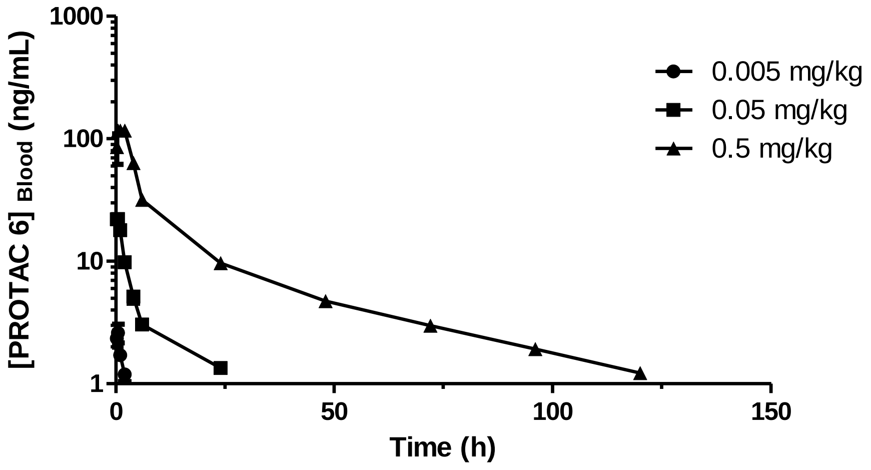

Supplementary Figure 5. Blood Concentration Data for PROTAC **6** associated with Figure 5a and 5b**.** Single SC dose at either 0.5mg/kg, 0.05mg/kg or 0.005mg/kg

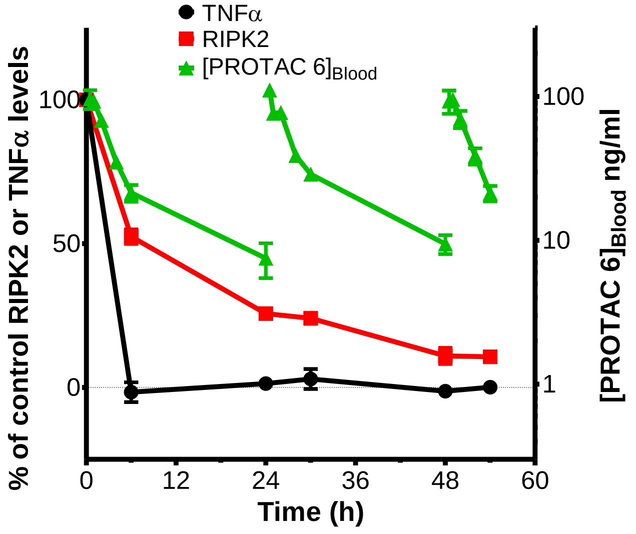

Supplementary Figure 6. The effect of dosing QD dosing of PROTAC **6** at 0.5mg/kg on both RIPK2 levels and TNFα levels following *ex vivo* L18-MDP challenge with associated drug levels.

Supplementary Figure 7. Additional Proteomic Plots for PROTAC 6

mPDP mature proteins after 6 h treatment

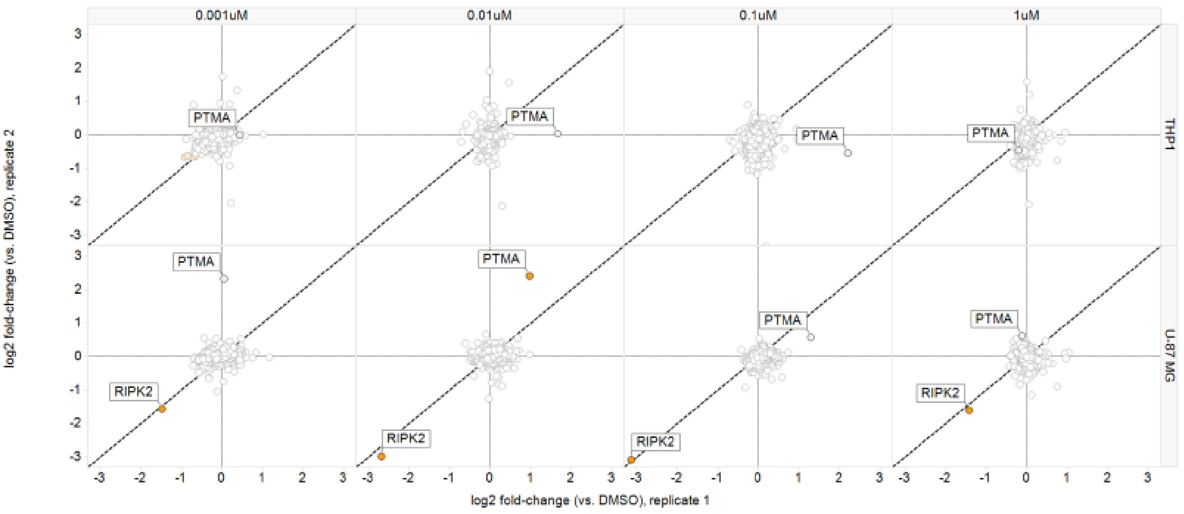

Scatter plot of mature proteins in THP1 or U-87 MG cells following 6 h treatment with 0.001 to 1 µM of PROTAC

mPDP nascent proteins after 6 h treatment

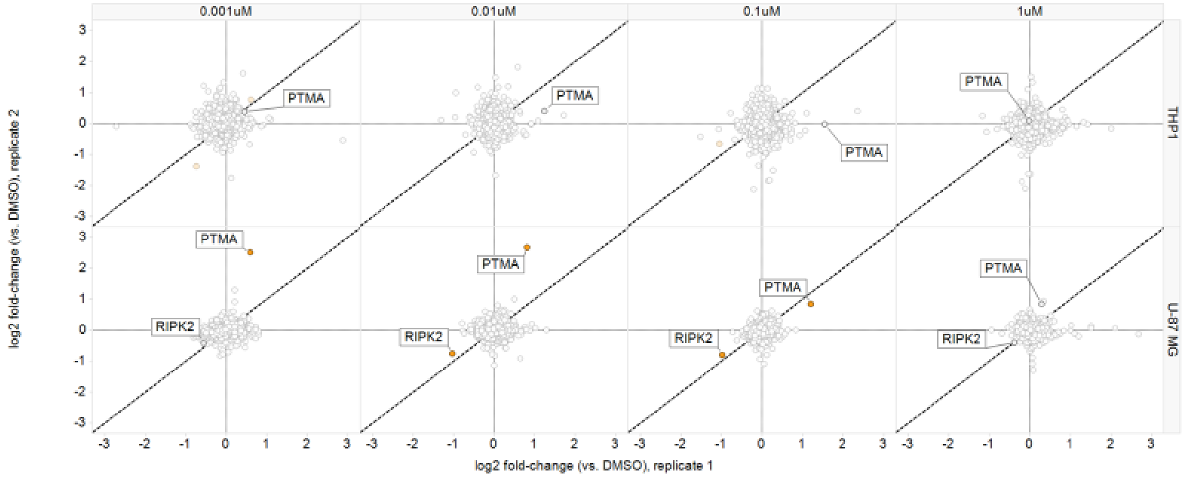

Scatter plot of nascent proteins in THP1 or U-87 MG cells following 6 h treatment with 0.001 to 1 μM of PROTAC

Kinase enrichment from mPDP mature proteins after 6 h treatment

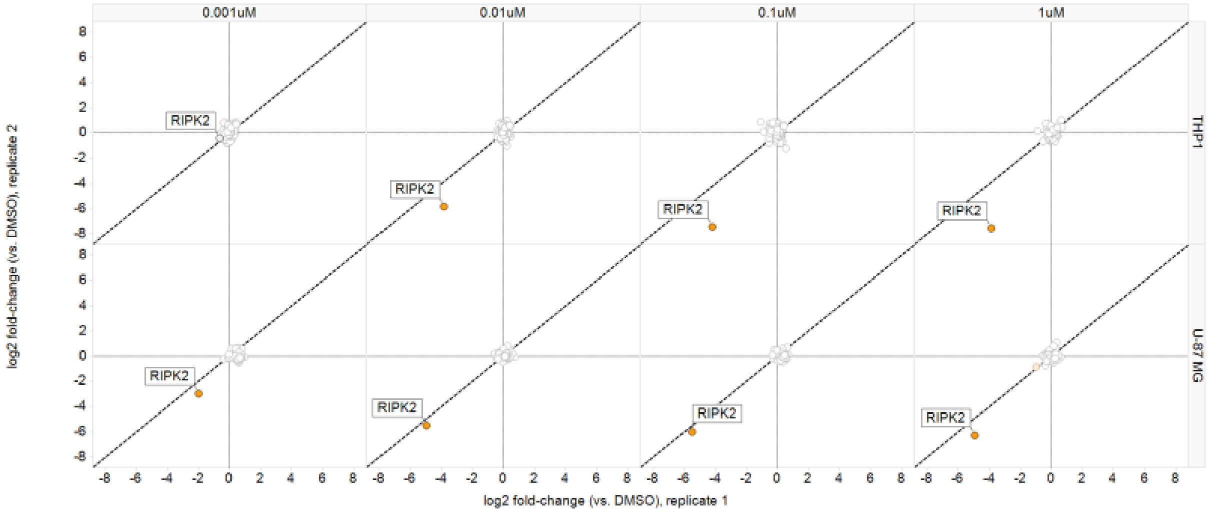

Scatter plot of mature proteins in THP1 or U-87 MG cells following 6 h treatment with 0.001 to 1 μM of PROTAC enriched for Kinases via a kinobeads affinity enrichment. Only Kinases are shown.

Kinase enrichment from mPDP nascent proteins after 6 h treatment

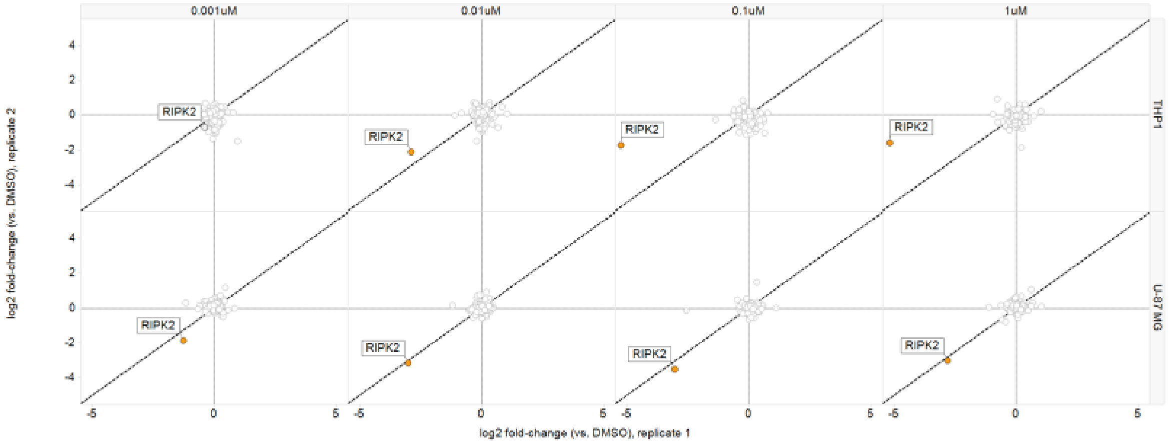

Scatter plot of nascent proteins in THP1 or U-87 MG cells following 6 h treatment with 0.001 to 1 µM of PROTAC enriched for Kinases via a kinobeads affinity enrichment. Only Kinases are shown.

mPDP mature proteins after 20 h treatment

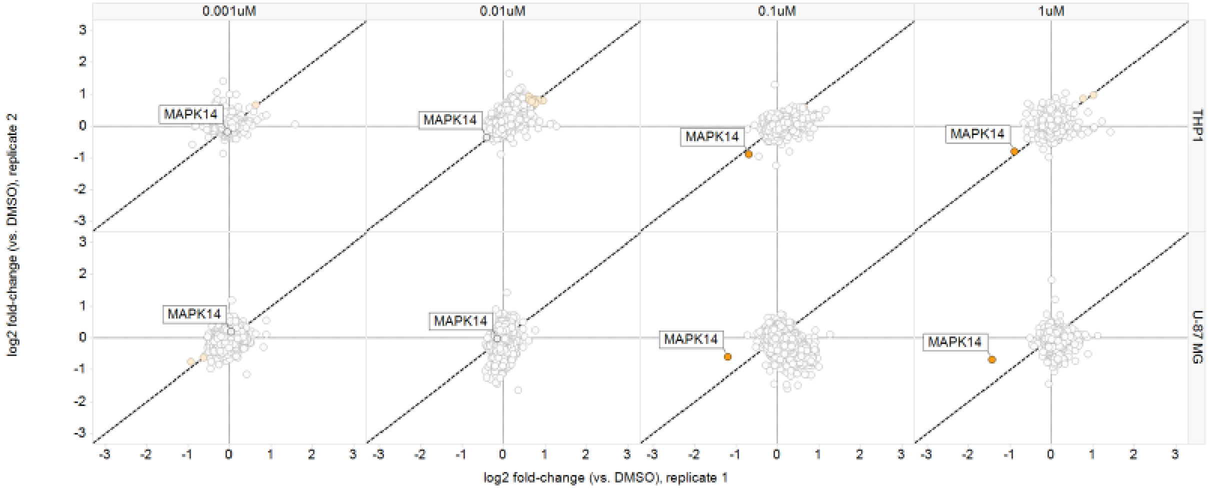

Scatter plot of mature proteins in THP1 or U-87 MG cells following 20 h treatment with 0.001 to 1 µM of PROTAC

mPDP nascent proteins after 20 h treatment

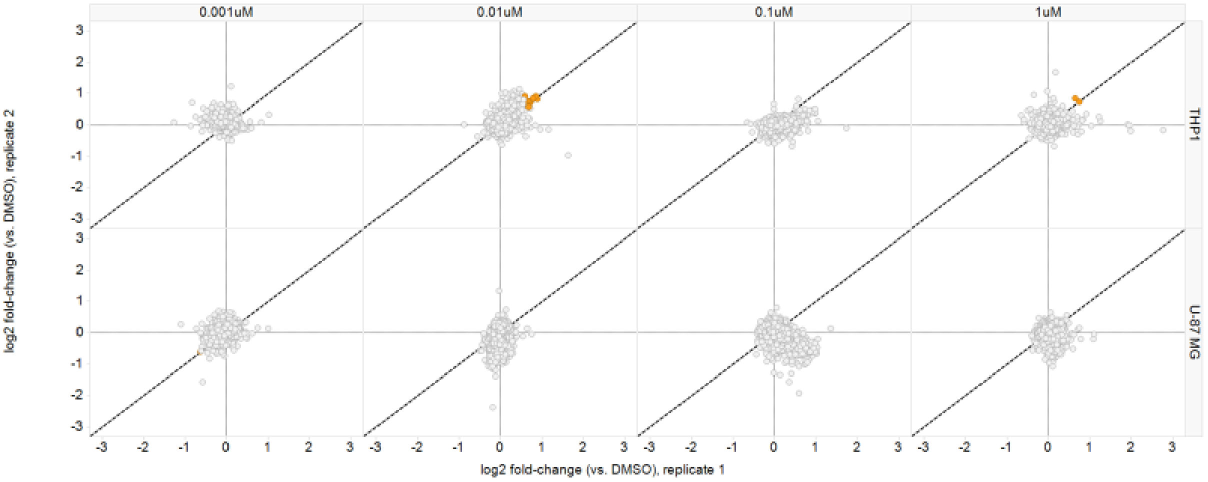

Scatter plot of nascent proteins in THP1 or U-87 MG cells following 20 h treatment with 0.001 to 1 µM of PROTAC

Kinase enrichment from mPDP mature proteins after 20 h treatment

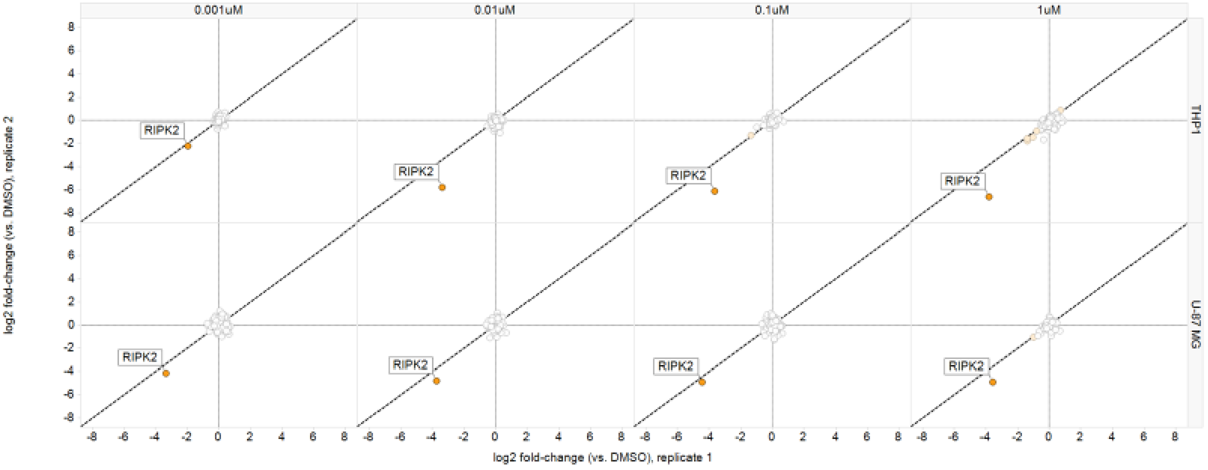

Scatter plot of mature proteins in THP1 or U-87 MG cells following 20 h treatment with 0.001 to 1 µM of PROTAC enriched for Kinases via a kinobeads affinity enrichment. Only Kinases are shown.

Kinase enrichment from mPDP nascent proteins after 20 h treatment

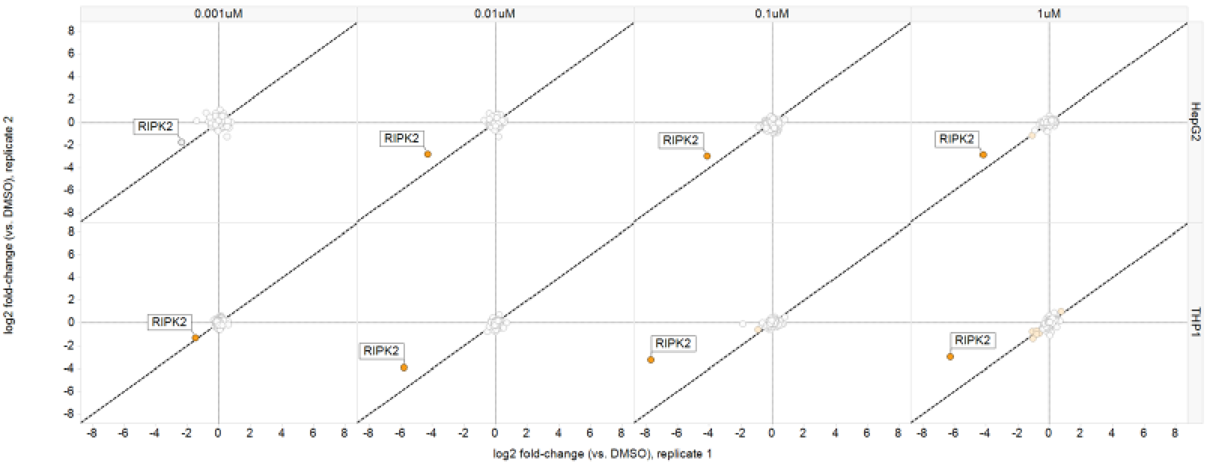

Scatter plot of nascent proteins in THP1 or U-87 MG cells following 20 h treatment with 0.001 to 1 µM of PROTAC enriched for Kinases via a kinobeads affinity enrichment. Only Kinases are shown.

Line plot for protein dynamics profiling

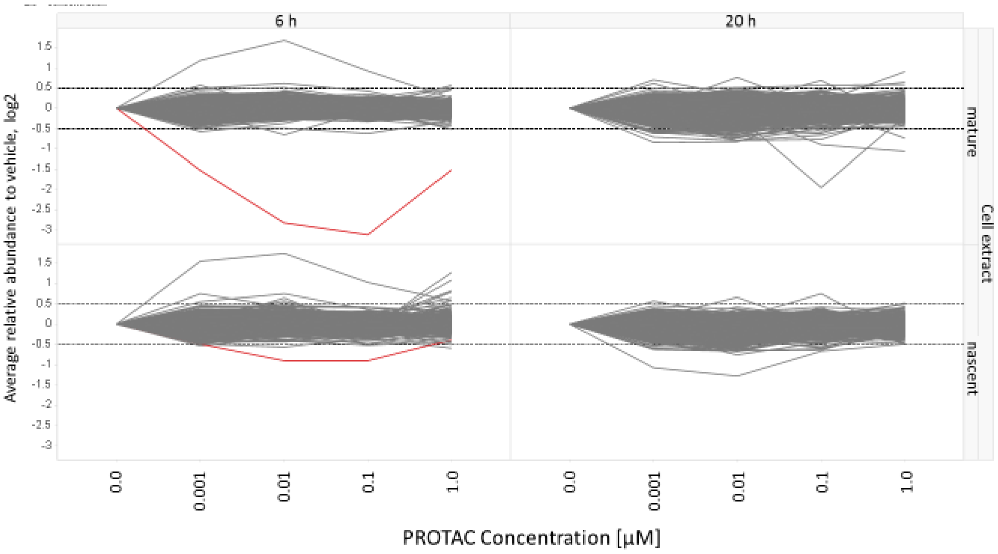

Line plot for protein dynamics profiling experiments separated for mature and nascent proteins following treatment of **5** in U-87 MG cells extract; RIPK2 shown in red.

Chemistry

General Experiment and Information

Organic solutions were dried over anhydrous Na_2SO_4, $MgSO_4$ or using a hydrophobic frit. TLC was performed on Merck 0.25 mm Kieselgel 60 F_{254} plates. Products were visualised under UV light and/or by staining with aqueous $KMnO_4$ solution.

LCMS analysis was conducted on either System A an Acquity UPLC CSH C_{18} column (2.1 mm × 50 mm ID, 1.7 µm packing diameter) eluting with 0.1% formic acid in water (solvent A), and 0.1 % formic acid in MeCN (solvent B), using the following elution gradient 0.0 – 1.5 min 3 – 100% B, 1.5 – 1.9 min 97% B, 1.9 – 2.0 min 97– 3% B, at a flow rate of 1 $mLmin^{-1}$ at 40 °C. The UV detection was an averaged signal from wavelength of 210 nm to 350 nm, and mass spectra were recorded on a mass spectrometer using alternate-scan electrospray positive and negative mode ionization (ES+ve and ES-ve); or System B an Acquity UPLC CSH C_{18} column (50 mm × 2.1 mm ID, 1.7 µm packing diameter) eluting with 10 mM ammonium bicarbonate in water adjusted to pH10 with ammonia solution (solvent A), and MeCN (solvent B) using the following elution gradient 0.0 – 1.5 min 3 – 95% B, 1.5 – 1.9 min 95% B, 1.9 – 2.0 min 97% B at a flow rate of 1 mL min^{-1} at 40 °C.

Column chromatography was performed on a Isolera™ purification system. The Isolera™ is an automated multi-user flash chromatography system, available from Biotage, which utilizes disposable, normal phase pre-packed silica, and C18 reverse phase cartridges (1 g to 340 g).

Purifications by mass-directed auto-preparative HPLC (MDAP) was conducted on either System A, an Xselect CSH C_{18} column (150 mm × 30 mm i.d. 5 µm packing diameter) at ambient temperature eluting with 0.1% formic acid in water (solvent A) and 0.1% formic acid in MeCN (solvent B), using an appropriate elution gradient over 15 or 25 min at a flow rate of 40 $mLmin^{-1}$ and detecting at 210 - 350 nm at room temperature. Mass spectra were recorded on Micromass ZMD mass spectrometer using electro spray positive and negative mode, alternate scans; or System B, an Xselect CSH C_{18} column (150 mm × 30 mm i.d. 5 µm packing diameter) at ambient temperature eluting with 10 mM ammonium bicarbonate in water adjusted to pH10 with ammonia solution (solvent A), and MeCN (solvent B), using an appropriate elution gradient over 15 or 25 min at a flow rate of 40 $mLmin^{-1}$. The software used was *MassLynx* 3.5 with *OpenLynx* and *FractionLynx* options.

ESI (+) high resolution mass spectra were obtained on a Waters XEVO G2-XS QTof hybrid quadrupole time-of-flight mass spectrometer, equipped with a Z-spray interface, over a mass range of 100 - 1200 Da. The elemental composition was calculated using MassLynx v4.1 for the $[M+H]^+$ or the $[M+2H]^{2+}/2$ and the mass error quoted as ppm.

Proton magnetic resonance spectra (^1H NMR) were recorded at 400 or 600 MHz, unless otherwise stated. The chemical shifts (δ) are expressed in ppm relative to internal solvent peaks. Carbon magnetic resonance spectra (^{13}C NMR) were recorded at 100 or 126 MHz.

Microwave reactions were carried out using a Biotage initiator microwave with a Biotage 60 sampler. The purity of all compounds screened in the biological assays was examined by LCMS analysis and was found to be ≥ 95%, unless otherwise specified. The chemical names were generated using ChemBioDraw Ultra v12 from CambridgeSoft.

<u>**Schemes**</u>

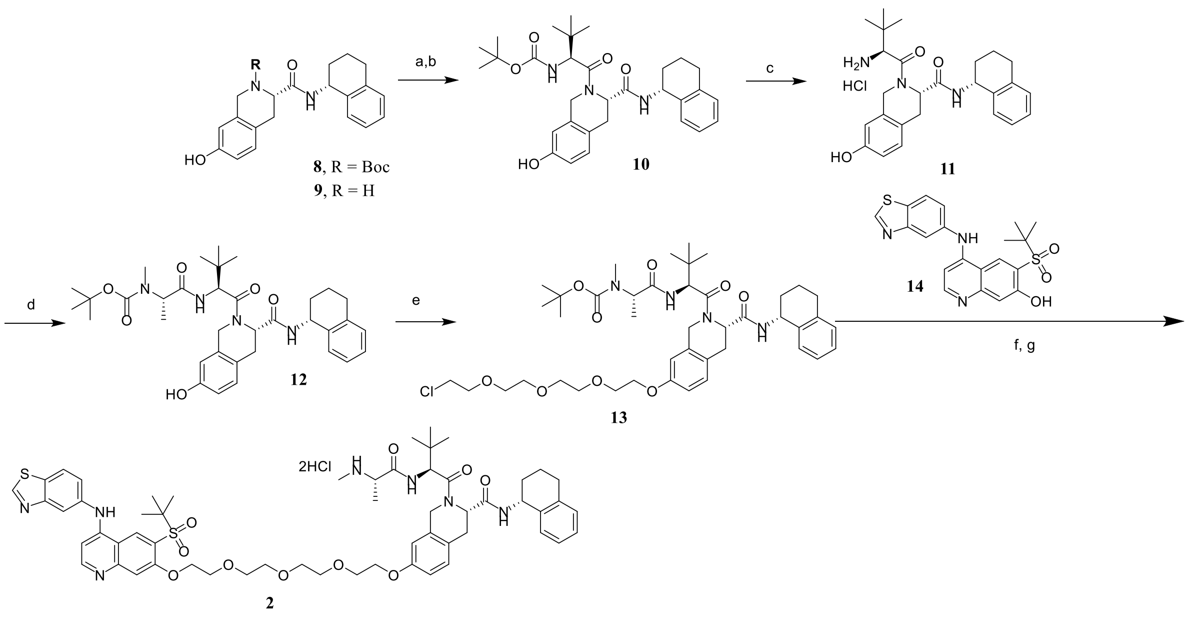

Scheme 1. Synthetic route for the preparation of PROTAC 2.

Reagents & Conditions: (a) 4 M HCl in dioxane, THF, rt, overnight, **9**, 94%; (b) (*S*)-*N*-Boc-2-amino-3,3-dimethylbutyric acid, HATU, DIPEA, DMF, rt, 1 h, 66%; (c) 4 M HCl in dioxane, THF, rt, overnight, 97%; (d) (*S*)-2-((*tert*-butoxycarbonyl)(methyl)amino)propanoic acid, HATU, DIPEA, DMF, rt, overnight, 77%; (e) 1-chloro-2-(2-(2-(2-chloroethoxy)ethoxy)ethoxy)ethane, K_2CO_3, DMF, 80 °C, overnight, 63%; (f) **14**, K_2CO_3, DMF, 105 °C, 8 h, 67%; (g) TFA, DCM, rt, 3 h, then 4M HCl in dioxane, 54%.

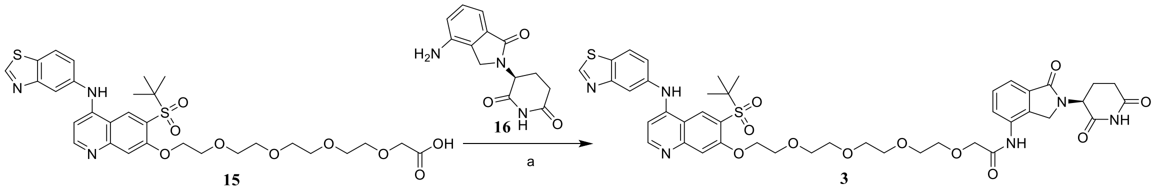

Scheme 2. **Synthetic route for the preparation of PROTAC 3.**

Reagents & Conditions: (a) **15**, **16**, HATU, DIPEA, DMF, rt, overnight, 46%.

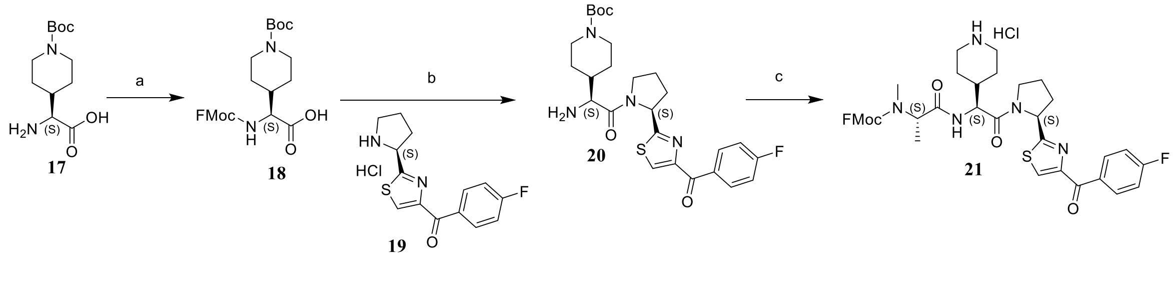

Scheme 3. Synthetic route to prepare IAP intermediate 21.

Reagents & Conditions: (a) 9-Fluorenylmethyl chloroformate, $NaHCO_3$, THF, H_2O, 0 °C, 30 min, then rt, 18 h, 98%; (b) i. **19**, HATU, DIPEA, DMF, rt, 18 h, ii. Piperidine, rt, 30 min, 53% over two steps; (c) i. (*S*)-2-((((9*H*-fluoren-9-yl)methoxy)carbonyl)(methyl)amino)- propanoic

acid, HATU, DIPEA, DMF, rt, 30 min, ii. 4 M HCl in dioxane, DCM, rt, 1 h, 61% over two steps.

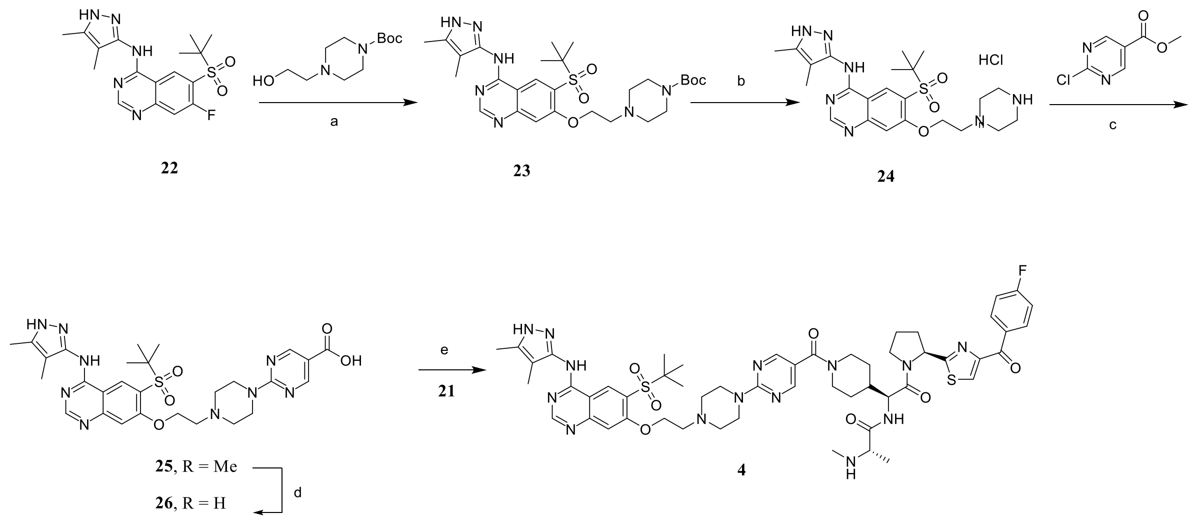

Scheme 4. Synthetic route for the preparation of PROTAC 4/5.

Reagents & Conditions: (a) NaH, 1,4 dioxane, 0 ºC, 1.5 h, then 90 ºC, 18 h, 50%; (b) 4 M HCl in dioxane, DCM, rt, 18 h, 98%; (c) DIPEA, NMP, 60 ºC, 2 h, 52%; (d) NaOH, THF, MeOH, H_2O, 18 h, then 4 M HCl in dioxane, 0.5 h, 100%; (e) i. **21**, HATU, *N*-methylmorpholine, DMF, rt, 30 min, ii. Piperidine, THF, rt, 1 h, 43% over two steps.

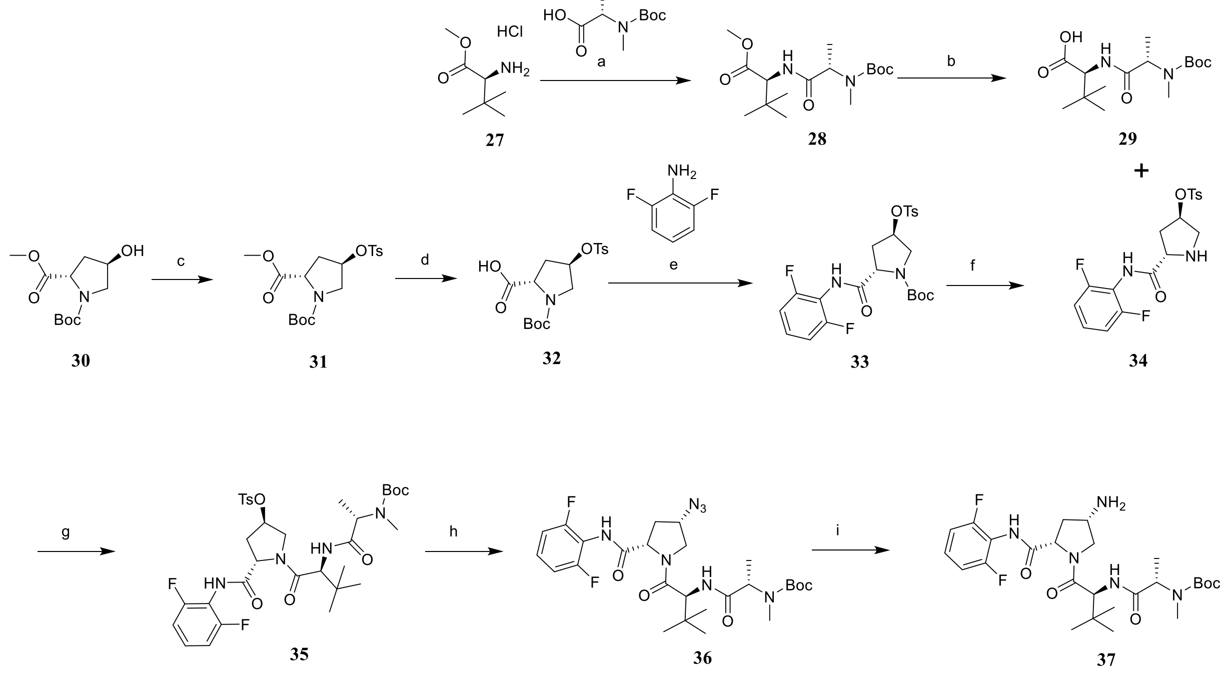

Scheme 5. Synthetic route to prepare IAP intermediate 37.

Reagents & Conditions: (a) Boc-*N*-methyl-*L*-alanine, HOBt, EDC, *N*-methylmorpholine, DCM, 0 ^{o}C, then rt, overnight, 5000 g, crude; (b) LiOH, H_2O, THF, MeOH, rt, 18 h, 4600 g, 99% yield over two steps, (c) TosCl, DMAP, DIPEA, DCM, rt, overnight, 6950 g, crude, (d) LiOH, H_2O, MeOH, rt, overnight, 5175 g , 82% yield over two steps, (e) 2,6-difluoroanilne, DCC, DCM, rt, overnight, 5065 g, crude, (f) TFA, DCM, rt, 3 h, 3665 g, 66% yield over two steps, (g) **29**, **34**, DCM, HOBt, EDC, - 20 ^{o}C, 1 h, then rt, overnight, 3400 g, 54% yield, (h) NaN_3, DMF, 80 ^{o}C, overnight, 1200 g, crude, (i) 10% Pd/C, MeOH, H_2, 3 h, 250 g, 9.5% yield over two steps.

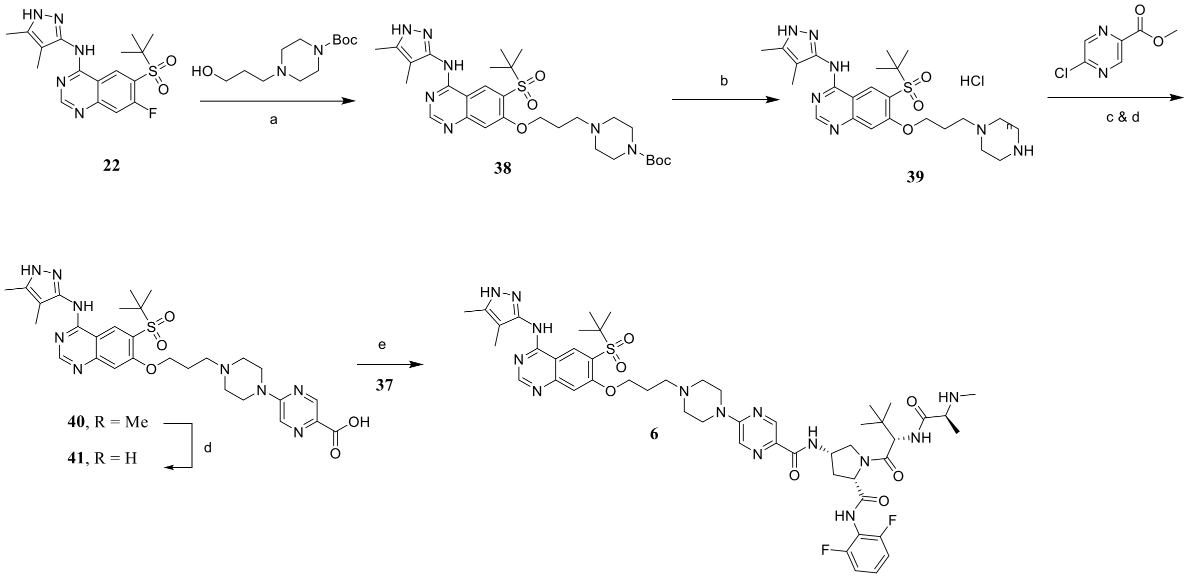

Scheme 8. Synthetic route for the preparation of PROTAC 6/7.

Reagents & Conditions: (a) NaH, 1,4 dioxane, 0 ^{o}C, 1.5 h, then 90 ^{o}C, 2 h, 86%; (b) 4 M HCl in dioxane, MeOH, 1.4-Dioxane, 50 ^{o}C, 2.5 h, 97%; (c) Et_3N, 1.4-Dioxane, 115 ^{o}C, 4 h, 67%; (d) DIPEA, H_2O, MeOH, 90 ^{o}C, 5 d, 91%; (e) i. **37**, HOBt, EDC, DIPEA, DMSO, rt, 20 h, ii. TFA, DCM, rt, 15 min, 0 ^{o}C, then rt, 1 h ,54% over two steps.

(*S*)-*tert*-Butyl 7-hydroxy-3-(((*R*)-1,2,3,4-tetrahydronaphthalen-1-yl)carbamoyl)-3,4-dihydroisoquinoline-2(1*H*)-carboxylate (8)

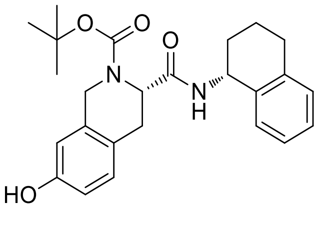

A mixture of (*S*)-2-(*tert*-butoxycarbonyl)-7-hydroxy-1,2,3,4-tetrahydroisoquinoline-3-carboxylic acid (1 g, 3.41 mmol) and (*R*)-1,2,3,4-tetrahydronaphthalen-1-amine (0.552 g, 3.8 mmol) in DMF (4 mL) was treated with DIPEA (1.8 mL, 10.2 mmol) and then with HATU (1.56 g, 4.1 mmol) and stirred at ambient temperature for 30 min. The mixture was treated with DCM (60 mL), saturated aqueous sodium bicarbonate (10 mL) and water (10 mL) and separated through a hydrophobic frit. The organic phase was evaporated to dryness and the

product was purified by chromatography on silica using a gradient elution from 0% to 100% EtOAc in cyclohexane to afford the title compound (1.18 g, 82% yield).
^{1}H NMR (400 MHz, DMSO-*d$_6$*) δ = 8.24 (br s, 1H), 8.03 - 7.79 (m, 1H), 7.15 - 7.05 (m, 1H), 7.04 - 6.87 (m, 3H), 6.64 (dd, *J* =8.0, 2.5 Hz, 1H), 6.60 - 6.40 (m, 2H), 4.93 - 4.57 (m, 1H), 4.52 - 4.25 (m, 3H), 3.13 - 2.84 (m, 2H), 2.79 - 2.58 (m, 2H), 1.95 - 1.52 (m, 4H), 1.50 - 1.29 (m, 9H); LCMS (Method A, UV, ES) RT = 1.10 min, $[M+H]^+$ = 423.5.

(*S*)-7-Hydroxy-*N*-((*R*)-1,2,3,4-tetrahydronaphthalen-1-yl)-1,2,3,4-tetrahydroisoquinoline-3-carboxamide, Hydrochloride (9)

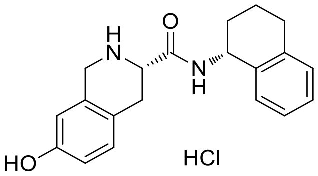

A solution of **8** (1.18 g, 2.8 mmol) in THF (10 mL) was treated with hydrochloric acid (4M in 1,4-dioxane) (10 mL, 40 mmol) and the mixture was stood at ambient temperature overnight. The mixture was removed of solvent *in vacuo* to afford the title compound (943 mg, 94% yield); ^{1}H NMR (400 MHz, DMSO-*d$_6$*) δ = 9.91 (br s, 1H), 9.69 - 9.18 (m, 2H), 8.96 (d, *J* = 8.5 Hz, 1H), 7.32 - 7.07 (m, 4H), 7.02 (d, *J* = 8.0 Hz, 1H), 6.70 (dd, *J* = 8.0, 2.5 Hz, 1H), 6.63 (d, *J* = 2.0 Hz, 1H), 5.24 - 4.89 (m, 1H), 4.37 - 4.16 (m, 2H), 4.12 - 4.00 (m, 1H), 3.18 (dd, *J* = 16.0, 4.5 Hz, 1H), 2.90 (dd, *J* = 16.0, 12.0 Hz, 1H), 2.84 - 2.64 (m, 2H), 2.00 - 1.63 (m, 4H); LCMS (Method A, UV, ES) RT = 0.58 min, $[M+H]^+$ = 323.4.

***tert*-Butyl ((*S*)-1-((*S*)-7-hydroxy-3-(((*R*)-1,2,3,4-tetrahydronaphthalen-1-yl)carbamoyl)-3,4-dihydroisoquinolin-2(1*H*)-yl)-3,3-dimethyl-1-oxobutan-2-yl)carbamate (10)**

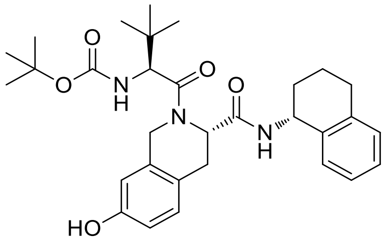

A mixture of **9** (933 mg, 2.6 mmol) and (*S*)-*N*-Boc-2-amino-3,3-dimethylbutyric acid (631 mg, 2.7 mmol) in DMF (10 mL) was treated with DIPEA (1.82 mL, 10.4 mmol) and then with HATU (1.19 g, 3.12 mmol) and stirred at ambient temperature for 1 h. The mixture was treated with DCM (80 mL), saturated aqueous sodium bicarbonate (10 mL) and water (10 mL) and separated through a hydrophobic frit. The organic phase was evaporated to dryness and the product was purified by chromatography on silica using a gradient elution from 0% to 100% EtOAc in cyclohexane to afford the title compound (923 mg, 66% yield); ^{1}H NMR (400 MHz, DMSO-*d$_6$*) δ = 9.45 - 9.07 (m, 1H), 8.26 - 7.91 (m, 1H), 7.21 - 6.85 (m, 5H), 6.75 - 6.43 (m, 3H), 5.27 - 4.71 (m, 2H), 4.68 - 4.07 (m, 3H), 3.02 - 2.82 (m, 2H), 2.79 - 2.59 (m, 2H), 1.96 - 1.47 (m, 4H), 1.41 - 1.20 (m, 9H), 1.07 - 0.87 (m, 9H); LCMS (Method A, UV, ES) RT = 1.27 min, $[M+H]^+$ = 536.6.

(*S*)-2-((*S*)-2-Amino-3,3-dimethylbutanoyl)-7-hydroxy-*N*-((*R*)-1,2,3,4-tetrahydronaphthalen-1-yl)-1,2,3,4-tetrahydroisoquinoline-3-carboxamide, Hydrochloride (11)

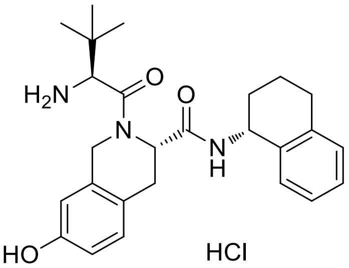

A solution of **10** (915 mg, 1.71 mmol) in THF (4 mL) was treated with hydrochloric acid (4M in 1,4-dioxane) (5 mL, 20 mmol) and then stirred at ambient temperature overnight. The mixture was evaporated to dryness to afford the title compound (780 mg, 97% yield); ^{1}H NMR (400 MHz, DMSO-*d*$_6$) δ = 8.39 (d, *J* = 9.0 Hz, 1H), 8.18 - 7.88 (m, 2H), 7.24 (d, *J* = 7.5 Hz, 1H), 7.19 - 6.94 (m, 4H), 6.82 (d, *J* = 2.0 Hz, 1H), 6.71 - 6.50 (m, 1H), 5.01 (d, *J* = 15.0 Hz, 1H), 4.96 - 4.74 (m, 1H), 4.64 - 4.45 (m, 2H), 4.39 (d, *J* = 15.0 Hz, 1H), 3.13 - 2.98 (m, 1H), 2.96 - 2.83 (m, 1H), 2.82 - 2.58 (m, 4H), 1.97 - 1.80 (m, 2H), 1.79 - 1.53 (m, 2H), 1.22 - 1.02 (m, 9H); LCMS (Method A, UV, ES) RT = 0.69 min, $[M+H]^+$ = 436.5.

***tert*-Butyl ((*S*)-1-(((*S*)-1-((*S*)-7-hydroxy-3-(((*R*)-1,2,3,4-tetrahydronaphthalen-1-yl)carbamoyl)-3,4-dihydroisoquinolin-2(1*H*)-yl)-3,3-dimethyl-1-oxobutan-2-yl)amino)-1-oxopropan-2-yl)(methyl)carbamate (12)**

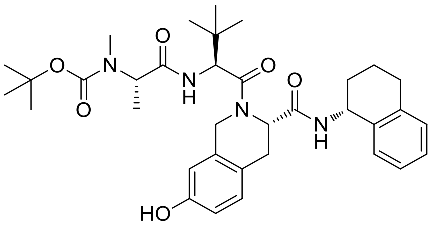

A mixture of **11** (770 mg, 1.63 mmol) and (S)-2-((tert-butoxycarbonyl)(methyl)amino) propanoic acid (332 mg, 1.63 mmol) in DMF (4 mL) was treated with DIPEA (1.14 mL, 6.5 mmol) and then with HATU (744 mg, 2.0 mmol) and stirred at ambient temperature overnight. The reaction mixture was partitioned between DCM (10 mL) and water (10 mL), the organic phase was collected and evaporated to dryness. The product was then purified by chromatography on silica using a gradient elution from 0% to 100% EtOAc in cyclohexane to afford the title compound (780 mg, 77% yield); ^{1}H NMR (400 MHz, DMSO-*d*$_6$) □ = 9.36 - 9.15 (m, 1H), 8.26 - 8.04 (m, 1H), 7.61 - 7.19 (m, 1H), 7.14 - 6.92 (m, 5H), 6.72 - 6.44 (m, 2H), 5.01 - 4.66 (m, 3H), 4.65 - 4.44 (m, 2H), 3.03 - 2.81 (m, 2H), 2.79 - 2.58 (m, 6H), 1.95 - 1.50 (m, 3H), 1.45 - 1.27 (m, 12H), 1.22 - 1.06 (m, 3H), 1.05 - 0.89 (m, 7H); LCMS (Method A, UV, ES) RT = 1.29 min, $[M+H]^+$ = 621.7.

***tert*-Butyl ((*S*)-1-(((*S*)-1-((*S*)-7-(2-(2-(2-(2-chloroethoxy)ethoxy)ethoxy)ethoxy)-3-(((*R*)-1,2,3,4-tetrahydronaphthalen-1-yl)carbamoyl)-3,4-dihydroisoquinolin-2(1H)-yl)-3,3-dimethyl-1-oxobutan-2-yl)amino)-1-oxopropan-2-yl)(methyl)carbamate (13)**

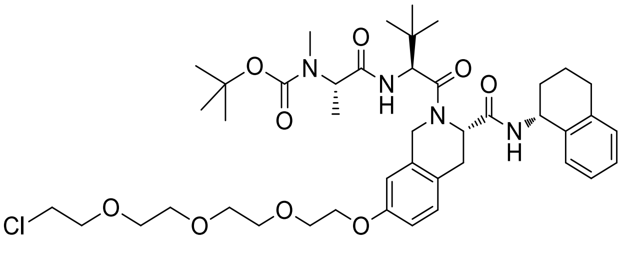

A solution of **12** (200 mg, 0.32 mmol) in DMF (4 mL) was treated with 1-chloro-2-(2-(2-(2-chloroethoxy)ethoxy)ethoxy)ethane (298 mg, 1.3 mmol) and potassium carbonate (134 mg, 1.0 mmol) and heated at 80 °C overnight. The product was subjected directly to purification by mass-directed automated preparative HPLC (Method A) to afford the title compound (165 mg, 63 % yield); ^{1}H NMR (400 MHz, DMSO-d_6) δ = 8.27 - 8.09 (m, 1H), 7.64 - 7.17 (m, 1H), 7.19 - 6.98 (m, 5H), 6.93 - 6.65 (m, 2H), 5.07 - 4.24 (m, 6H), 4.15 - 3.96 (m, 2H), 3.83 - 3.63 (m, 6H), 3.63 - 3.43 (m, 9H), 3.07 - 2.88 (m, 2H), 2.80 - 2.55 (m, 4H), 1.98 - 1.51 (m, 4H), 1.52 - 1.33 (m, 9H), 1.24 - 1.06 (m, 3H), 1.07 - 0.87 (m, 9H); LCMS (Method A, UV, ES) RT = 1.44 min, $[M+H]^+$ = 816.4.

tert-Butyl ((_S_)-1-(((_S_)-1-((_S_)-7-(2-(2-(2-(2-((4-(benzo[_d_]thiazol-5-ylamino)-6-(_tert_-butylsulfonyl)quinolin-7-yl)oxy)ethoxy)ethoxy)ethoxy)ethoxy)-3-(((_R_)-1,2,3,4-tetrahydronaphthalen-1-yl)carbamoyl)-3,4-dihydroisoquinolin-2(1_H_)-yl)-3,3-dimethyl-1-oxobutan-2-yl)amino)-1-oxopropan-2-yl)(methyl)carbamate (2-Boc)

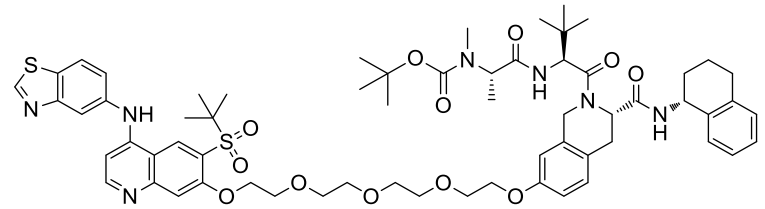

A mixture of 4-(benzo[*d*]thiazol-5-ylamino)-6-(*tert*-butylsulfonyl)quinolin-7-ol (**14**) (61 mg, 0.15 mmol), **13** (80 mg, 0.098 mmol) and potassium carbonate (41 mg, 0.29 mmol) in DMF (1.5 mL) was heated at 105 °C for 8 h. The crude product was subjected directly to purification by mass-directed automated preparative HPLC (Method A) to afford the title compound (78 mg, 67 % yield); ^{1}H NMR (400 MHz, $CDCl_3$) δ = 9.14 - 9.07 (m, 1H), 8.93 - 8.81 (m, 1H), 8.44 - 8.30 (m, 2H), 8.16 - 8.00 (m, 2H), 7.82 (d, J = 5.5 Hz, 1H), 7.54 - 7.35 (m, 1H), 7.17 - 6.76 (m, 7H), 6.72 - 6.16 (m, 3H), 5.20 - 4.95 (m, 3H), 4.94 - 4.66 (m, 1H), 4.65 - 4.47 (m, 1H), 4.44 - 4.24 (m, 3H), 4.04 (q, J = 5.0 Hz, 2H), 3.99 - 3.90 (m, 2H), 3.88 - 3.79 (m, 2H), 3.80 - 3.64 (m, 10H), 3.53 - 3.16 (m, 1H), 3.01 - 2.83 (m, 1H), 2.78 - 2.60 (m, 5H), 2.00 - 1.83 (m, 1H), 1.82 - 1.63 (m, 3H), 1.51 - 1.44 (m, 9H), 1.41 - 1.33 (m, 9H), 1.07 - 0.97 (m, 6H), 0.95 (s, 3H); LCMS (Method B, UV, ES) RT = 1.48 min, $[M+H]^+$ = 1193.1.

(_S_)-7-(2-(2-(2-(2-((4-(benzo[_d_]thiazol-5-ylamino)-6-(_tert_-butylsulfonyl)quinolin-7-yl)oxy)ethoxy)ethoxy)ethoxy)ethoxy)-2-((_S_)-3,3-dimethyl-2-((_S_)-2-(methylamino)propanamido)butanoyl)-_N_-((_R_)-1,2,3,4-tetrahydronaphthalen-1-yl)-1,2,3,4-tetrahydroisoquinoline-3-carboxamide, 2Hydrochloride (2)

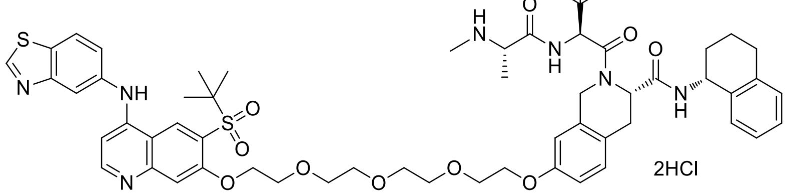

Trifluoroacetic acid (0.26 mL, 3.35 mmol) was added to a solution of **2-Boc** (80 mg, 0.067 mmol) in DCM (0.75 mL) and the reaction mixture was stirred at ambient temperature for 3 h. The solution was evaporated to dryness and the residual product was subjected to purification by mass-directed automated preparative HPLC (Method A). The recovered material was treated with 4M HCl in 1,4-dioxane (2 mL), evaporated and dried under vacuum

to afford the title compound (42 mg, 54 % yield); ^{1}H NMR (400 MHz, MeOD-*d*4) δ = 9.40 (s, 1H), 9.19 (s, 1H), 8.46 - 8.23 (m, 3H), 8.17 (d, *J* = 2.0 Hz, 1H), 7.60 (dd, *J*= 8.0, 2.0 Hz, 1H), 7.50 (s, 1H), 7.25 - 6.93 (m, 5H), 6.90 - 6.63 (m, 3H), 5.17 - 5.05 (m, 1H), 5.03 - 4.84 (m, 2H), 4.76 - 4.54 (m, 2H), 4.50 - 4.37 (m, 2H), 4.15 - 4.04 (m, 2H), 4.01 - 3.89 (m, 3H), 3.87 - 3.79 (m, 2H), 3.77 - 3.65 (m, 8H), 3.08 - 2.99 (m, 1H), 2.84 - 2.69 (m, 2H), 2.66 (s, 3H), 2.01 - 1.55 (m, 5H), 1.45 (br. s, 12H), 1.39 (d, *J* = 7.0 Hz, 2H), 1.20 - 1.00 (m, 9H), note. additional peaks observed due to rotamers; ^{13}C NMR (151 MHz, DMSO-d_6) δ = 170.62, 169.61, 168.60, 158.49, 157.26, 154.11, 137.71, 136.92, 136.14, 128.61, 128.27, 126.48, 125.69, 125.13, 113.22, 112.10, 110.98, 100.87, 70.09, 61.33, 56.05, 55.60, 54.70, 46.95, 39.76, 35.49, 31.11, 28.86, 26.62, 23.70, 16.06; HRMS (TOFMS ES+, *m/z*) exact mass $C_{58}H_{73}N_7O_{10}S_2$ 1091.4861, found 1092.4913; LCMS (Method B, UV, ES) RT = 1.31 min, $[M+H]^+$ = 1093.7.

(*S*)-14-((4-(Benzo[*d*]thiazol-5-ylamino)-6-(tert-butylsulfonyl)quinolin-7-yl)oxy)-*N*-(2-(2,6-dioxopiperidin-3-yl)-1-oxoisoindolin-4-yl)-3,6,9,12-tetraoxatetradecan-1-amide (3)

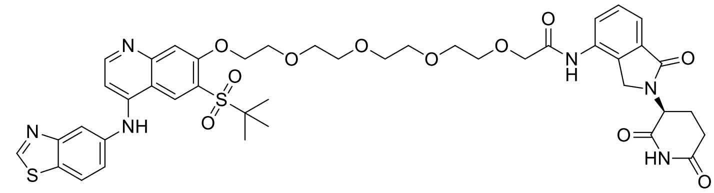

A mixture of 14-((4-(benzo[*d*]thiazol-5-ylamino)-6-(*tert*-butylsulfonyl)quinolin-7-yl)oxy)-3,6,9,12-tetraoxatetradecan-1-oic acid (27 mg, 0.042 mmol) (Described in *Nat. Chem. Biol.* **2015**, 11, 611.) and (*S*)-3-(4-amino-1-oxoisoindolin-2-yl)piperidine-2,6-dione (10.81 mg, 0.042 mmol) in DMF (0.7 mL) was treated with DIPEA (0.029 mL, 0.167 mmol) and then with HATU (19 mg, 0.05 mmol). After stirring at ambient temperature overnight, the crude mixture was subjected to purification by mass-directed automated preparative HPLC (Method A) to afford the title compound (17 mg, 46% yield); ^{1}H NMR (400 MHz, MeOD-d_4) δ = 9.32 (s, 1H), 8.96 (s, 1H), 8.43-8.32 (m, 2H), 8.17 (d, *J* = 8.5 Hz, 1H), 8.10 (d, *J* = 2.0 Hz, 1H), 7.71 (dd, J = 8.0, 1.0 Hz, 1H), 7.65-7.53 (m, 2H), 7.48 (t, *J* = 8.0 Hz, 1H), 7.40 (s, 1H), 6.88 (d, *J* = 6.0 Hz, 1H), 5.48 (s, 2H), 5.15 (dd, *J* = 13.0, 5.0 Hz, 1H), 4.51 (d, *J* = 2.9 Hz, 2H), 4.37-4.26 (m, 2H), 4.19 (s, 2H), 3.90 (dd, *J* = 5.0, 4.0 Hz, 2H), 3.82-3.70 (m, 4H), 3.60-3.69 (m, 6H), 3.59-3.53 (m, 2H), 2.98-2.68 (m, 3H), 2.58-2.37 (m, 1H), 2.25-2.09 (m, 1H), 1.41 (s, 9H); HRMS (TOFMS ES+, m/z) exact mass $C_{43}H_{48}N_6O_{11}S_2$ 888.2822, found 889.2910; LCMS (Method B, UV, ES) RT = 0.96 min, [M+H]+ = 889.2.

(*S*)-2-((((9*H*-Fluoren-9-yl)methoxy)carbonyl)amino)-2-(1-(*tert*-butoxycarbonyl)piperidin-4-yl)acetic acid (18)

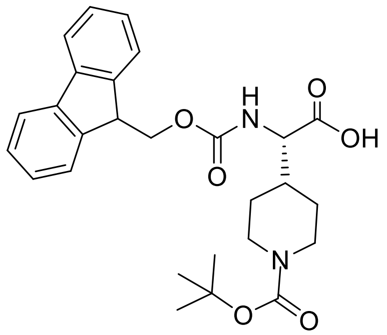

To a suspension of (*S*)-2-amino-2-(1-(tert-butoxycarbonyl)piperidin-4-yl)acetic acid (0.51 g, 1.97 mmol) in THF (7.5 mL) and Water (7.5 mL) at 0 °C, was added 9-fluorenylmethyl

chloroformate (0.51 g, 1.97 mmol) and sodium carbonate (0.209 g, 1.974 mmol). The resulting mixture was stirred at 0 °C for 30 min, and then stirred at ambient temperature overnight. The reaction mixture was diluted with EtOAc (40 mL) and the pH was adjusted to around 2-3 with 1M HCl. The organic layer was separated, and the aqueous layer was extracted with additional EtOAc (2 x 40 mL). The combined organic layers were washed with brine (50 mL), dried using a hydrophobic frit and concentrated under reduced pressure to afford the title compound (930 mg, 98% yield); ^{1}H NMR (400 MHz, DMSO-d_6) δ = 7.88 (d, *J* = 7.3 Hz, 2H), 7.67 (d, *J* = 7.3 Hz, 2H), 7.46 - 7.24 (m, 4H), 6.34 (br s, 1H), 4.36 - 4.16 (m, 2H), 3.99 - 3.68 (m, 2H), 3.48 (br s, 1H), 3.40 - 3.25 (m, 4 H obscured by water peak), 1.93 - 1.71 (m, 1H), 1.63 - 1.45 (m, 1H), 1.44 - 1.21 (m, 10H), 1.12 - 0.89 (m, 1H); LCMS (Method B, UV, ES) RT = 1.27 min, [M+Na]+ = 503.2, $[M-H]^-$ = 479.3.

***tert*-Butyl 4-((*S*)-1-amino-2-((*S*)-2-(4-(4-fluorobenzoyl)thiazol-2-yl)pyrrolidin-1-yl)-2-oxoethyl)piperidine-1-carboxylate, Formic acid salt (20)**

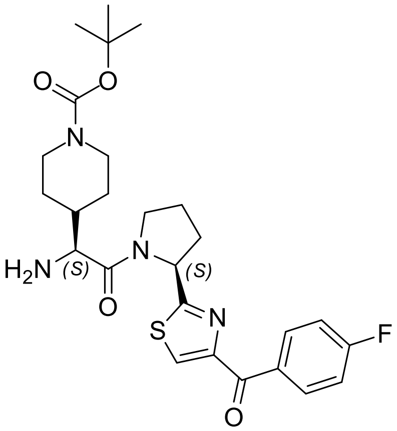

HATU (511 mg, 1.34 mmol) was added to a mixture of **18** (565 mg, 1.18 mmol), (*S*)-(4-fluorophenyl)(2-(pyrrolidin-2-yl)thiazol-4-yl)methanone, Hydrochloride (350 mg, 1.119 mmol, obtained as described in WO2008016893A1) and DIPEA (0.59 mL, 3.36 mmol) in DMF (3 mL). The reaction was stirred at ambient temperature for 18 h. Piperidine (0.33 mL, 3.36 mmol) was then added, and the mixture was stirred for an additional 30 min. The product was subjected directly to purification by reverse phase chromatography (60 g C18 column) using a gradient elution from 5% to 60% acetonitrile in water with a 0.1% formic acid modifier to afford the title compound (335 mg, 53% yield); ^{1}H NMR (400 MHz, DMSO-d_6) □ = 8.56 (s, 0.25H, rotamer), 8.48 (s, 0.75H, rotamer), 8.27 - 8.20 (m, 2H), 8.18 (s, 1H), 7.39 (t, *J* = 9.0 Hz, 2H), 5.63 - 5.35 (m, 1H), 4.04 - 3.81 (m, 4H), 3.80 - 3.66 (m, 3H), 3.45 (d, *J* = 6.0 Hz, 2H), 2.40 - 2.14 (m, 2H), 2.10 - 1.96 (m, 2H), 1.75 - 1.56 (m, 2H), 1.56 - 1.45 (m, 1H), 1.38 (br s, 9H), 1.33 - 1.18 (m, 1H), 1.16 - 0.97 (m, 1H); LCMS (Method A, UV, ES) RT = 0.76 min, $[M+H]^+$ = 517.3.

(9*H*-Fluoren-9-yl)methyl ((*S*)-1-(((*S*)-2-((*S*)-2-(4-(4-fluorobenzoyl)thiazol-2-yl)pyrrolidin-1-yl)-2-oxo-1-(piperidin-4-yl)ethyl)amino)-1-oxopropan-2-yl)(methyl)carbamate, Hydrochloride (21)

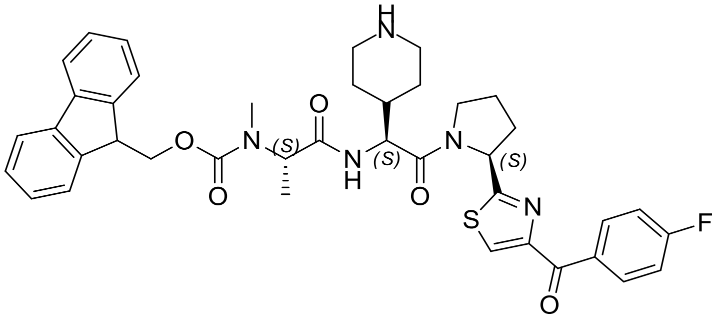

HATU (272 mg, 0.714 mmol) was added to a mixture of (*S*)-2-((((9H-fluoren-9-yl)methoxy)carbonyl)(methyl)amino)propanoic acid (232 mg, 0.714 mmol) **20**, (335 mg, 0.595 mmol) and DIPEA (0.312 mL, 1.79 mmol) in DMF (3 mL). After stirring at ambient temperature for 30 min, the reaction mixture was partitioned between EtOAc (80 mL) and water (50 mL). The organic layer was separated, washed with water (50 mL), brine (50 mL), dried using a hydrophobic frit, and concentrated under reduced pressure. The crude material was loaded in DCM and purified on a 50g silica column, using a gradient of 0-100% EtOAc in cyclohexane, followed by 0-10% methanol in DCM. The appropriate fractions were combined and evaporated under reduced pressure. The purified material was taken up in DCM (5 mL), treated with 4M HCl in Dioxane (0.74 mL, 2.98 mmol), and stirred at ambient temperature for 1 h. The solvent was removed under reduced pressure, and the residual acid was removed by addition and evaporation of toluene (2 x 20 mL) to afford the title compound (275 mg, 61% yield) ; ^{1}H NMR (400 MHz, DMSO-d_6) δ = 8.94 (br d, *J* = 10.0 Hz, 1H), 8.72 (br d, *J* = 10.0 Hz, 1H), 8.48 (s, 1H), 8.30 - 8.09 (m, 2H), 7.95 - 7.83 (m, 2H), 7.64 (d, *J* = 7.0 Hz, 2H), 7.48 - 7.27 (m, 6H), 5.37 (br s, 1H), 4.63 (q, *J* = 7.0 Hz, 1H), 4.47 (t, *J* = 8.0 Hz, 1H), 4.41 - 4.15 (m, 2H), 3.79 (t, *J* = 7.0 Hz, 2H), 3.19 (br s, 1H), 3.04 (br d, *J* = 11.0 Hz, 1H), 2.80 (br s, 3H), 2.76 - 2.56 (m, 2H), 2.37 - 2.21 (m, 1H), 2.20 - 2.09 (m, 1H), 2.09 - 1.94 (m, 3H), 1.81 - 1.68 (m, 2H), 1.57 - 1.33 (m, 2H), 1.25 (br s, 3H); ^{13}C NMR (DMSO-d_6, 151 MHz) □ = 184.6, 172.8, 171.4, 169.4, 164.8, 155.4, 152.4, 143.7, 140.7, 133.4, 133.0, 129.9, 127.6, 127.1, 125.0, 120.1, 115.4, 66.8, 58.3, 54.1, 53.8, 47.1, 46.7, 42.5, 35.5, 31.6, 30.0, 25.2, 24.3,24.0, 14.8; LCMS (Method A, UV, ES) RT = 0.90 min, $[M+H]^+$ = 724.3.

***tert*-Butyl 4-(2-((6-(*tert*-butylsulfonyl)-4-((4,5-dimethyl-1*H*-pyrazol-3-yl)amino)quinazolin-7-yl)oxy)ethyl)piperazine-1-carboxylate (23)**

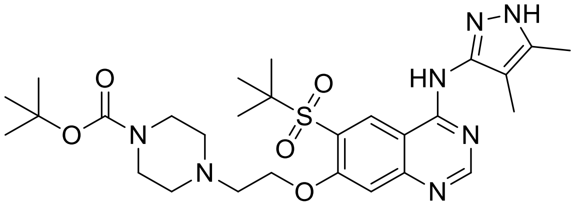

Under nitrogen, an ice-cooled mixture of 6-(tert-butylsulfonyl)-*N*-(4,5-dimethyl-1*H*-pyrazol-3-yl)-7-fluoroquinazolin-4-amine, **22** (obtained as described in WO2014128622A1) (10 g, 26.5 mmol) and tert-butyl 4-(2-hydroxyethyl)piperazine-1-carboxylate (18.31 g, 79 mmol) in 1,4-Dioxane (300 mL) was treated cautiously with sodium hydride 60% (6.36 g of a 60% w/w dispersion in mineral oil, 159 mmol) and stirred for 1.5 h. The mixture was then heated at 100 °C for 16 h and cooled back to 0 °C. The mixture was carefully treated with saturated ammonium chloride solution (30 mL) and water (100 mL). The organic portion was removed under reduced pressure, and the aqueous layer was extracted with EtOAc (200 mL x 2). The combined organic layers were then dried using a hydrophobic frit and concentrated under

reduced pressure. The solid residue was loaded preabsorbed on Florisil and purified on a 340 g silica column, using a gradient of 5-15% MeOH in DCM. The appropriate fractions were combined and evaporated under reduced pressure to afford the title compound (7.8 g, 50% yield); ^{1}H NMR (400 MHz, MeOD-d_4) δ = 8.91 (s, 1H), 8.43 (br s, 1H), 7.35 (s, 1H), 4.39 (t, J = 5.5 Hz, 2H), 3.50 - 3.40 (m, 4H), 2.93 (t, J = 5.5 Hz, 2H), 2.67 - 2.56 (m, 4H), 2.27 (s, 3H), 1.87 (br s, 3H), 1.51 - 1.44 (m, 9H), 1.41 (s, 9H); LCMS (HpH Method, UV, ES) RT = 1.05 min, $[M+H]^+$ = 588.2.

6-(*tert*-Butylsulfonyl)-*N*-(4,5-dimethyl-1*H*-pyrazol-3-yl)-7-(2-(piperazin-1-yl)ethoxy)quinazolin-4-amine, 3 Hydrochloride (24)

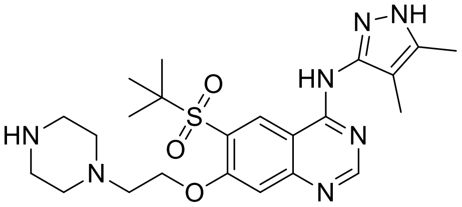

A solution of **23** (7.81 g, 13.29 mmol) in DCM (100 mL) was treated with hydrochloric acid (4M in 1,4-dioxane) (20 mL, 80 mmol) and the mixture was stirred at ambient temperature for 18 h. The mixture was removed of solvent *in vacuo* and triturated with diethyl ether to afford the title compound (7.77 g, 98% yield); ^{1}H NMR (400 MHz, MeOD-d_4) δ = 9.13 (br s, 1H), 8.88 (s, 1H), 7.55 (s, 1H), 4.97 (t, J = 4.5 Hz, 2H), 4.01 - 3.91 (m, 4H), 3.78 - 3.70 (m, 4H), 3.33 (2H Obscurred not observed); 2.38 (s, 3H), 2.04 (s, 3H), 1.47 (s, 9H); LCMS, Method B, UV, ES) RT = 0.78 min, $[M+H]^+$ = 488.6.

Methyl 2-(4-(2-((6-(*tert*-butylsulfonyl)-4-((4,5-dimethyl-1*H*-pyrazol-3-yl)amino)quinazolin-7-yl)oxy)ethyl)piperazin-1-yl)pyrimidine-5-carboxylate (25)

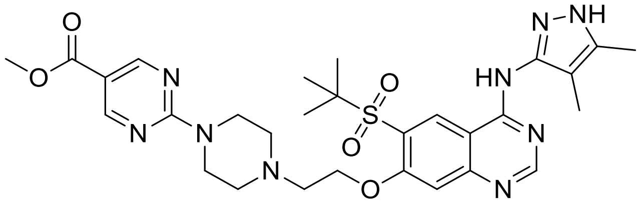

A mixture of **24** (1.52g, 3.11 mmol), methyl 2-chloropyrimidine-5-carboxylate (1.08g, 6.23 mmol) and DIPEA (2.18 mL, 12.5 mmol) in NMP (6 mL) was heated in a Biotage Initiator microwave at 60 °C for 2 h. The reaction mixture was cooled, loaded directly and purified by reverse phase chromatography (120 g C18 column) using a gradient elution from 5% to 70% acetonitrile in water (+ 0.01% ammonium bicarbonate modifier) to afford the title product (1.63 g, 52% yield); ^{1}H NMR (400 MHz, MeOD-d_4) δ = 8.91 (s, 1H), 8.81 (s, 2H), 8.44 (br s, 1H), 7.36 (s, 1H), 4.43 (t, J = 5.5 Hz, 2H), 4.01 - 3.90 (m, 4H), 3.86 (s, 3H), 2.97 (t, J = 5.5 Hz, 2H), 2.78 - 2.69 (m, 4H), 2.27 (s, 3H), 1.88 (br s, 3H), 1.42 (s, 9H); LCMS (Method B, UV, ES) RT = 1.01 min, $[M+H]^+$ = 624.2.

2-(4-(2-((6-(tert-butylsulfonyl)-4-((4,5-dimethyl-1H-pyrazol-3-yl)amino)quinazolin-7-yl)oxy)ethyl)piperazin-1-yl)pyrimidine-5-carboxylic acid, 3 Hydrochloride (26)

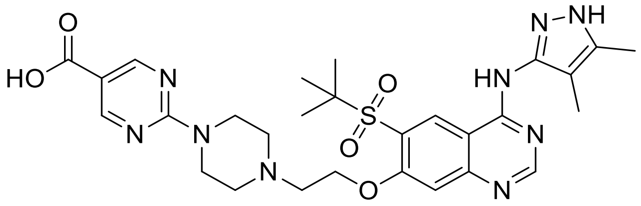

NaOH (1.7 mL, 3.4 mmol) was added to a solution of **25** (720 mg, 1.15 mmol) in THF (8 mL) and MeOH (4 mL), and the reaction was stirred at room temperature for 18 h. The reaction was concentrated to remove the organic solvent, then taken up in DCM (6 mL) and acidified with 4M HCl in Dioxane (4.5 mL, 18 mmol). After stirring at room temperature for 0.5 h, the volatiles were removed under reduced pressure, and dried under high vacuum to afford the title product (827 mg, quantitative yield, note. contaminated with 3 eq. of NaCl); ^{1}H NMR (400 MHz, MeOD-*d*$_4$) δ = 8.92 (s, 1H), 8.82 (s, 2H), 8.43 (s, 1H), 7.36 (s, 1H), 4.46 (t, *J* = 5.5 Hz, 2H), 3.98 (t, *J* = 5.0 Hz, 4H), 3.06 (t, *J* = 5.5 Hz, 2H), 2.82 (t, *J* = 5.0 Hz, 4H), 2.27 (s, 3H), 1.89 (s, 3H), 1.42 (s, 9H); LCMS (HpH Method, UV, ES) RT = 0.66 min, [M+H]$^+$ = 610.2.

(*S*)-*N*-((*S*)-1-(1-(2-(4-(2-((6-(*tert*-Butylsulfonyl)-4-((4,5-dimethyl-1*H*-pyrazol-3-yl)amino)quinazolin-7-yl)oxy)ethyl)piperazin-1-yl)pyrimidine-5-carbonyl)piperidin -4-yl)-2-((S)-2-(4-(4-fluorobenzoyl)thiazol-2-yl)pyrrolidin-1-yl)-2-oxoethyl)-2-(methylamino)propanamide (4)

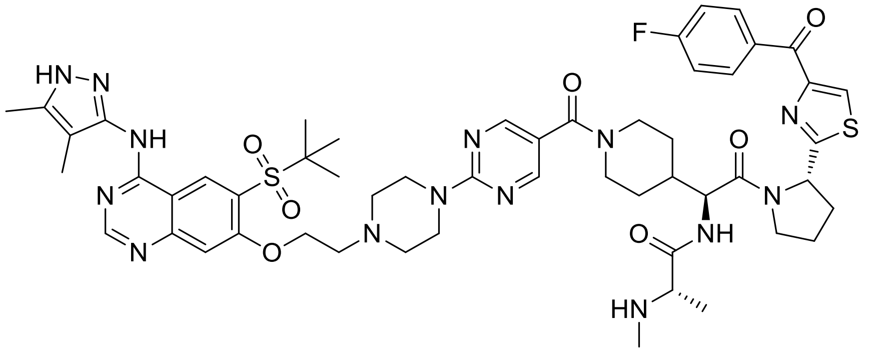

HATU (25 mg, 0.066 mmol) was added to a mixture of **21** (40 mg, 0.055 mmol), **26** (56 mg, 0.055 mmol) and DIPEA (0.06 mL, 0.33 mmol) in DMF (0.8 mL). The reaction was stirred at ambient temperature for 30 min. Piperidine (0.11 mL, 1.1 mmol) was added, and the mixture was stirred for an additional 1 h before being subjected directly to purification by mass-directed automated preparative HPLC (Method B). The appropriate fractions were combined and the solvent was concentrated under reduced pressure to afford the title compound (26 mg, 43% yield); ^{1}H NMR (400 MHz, MeOD-*d*4) δ = 8.91 (s, 1H), 8.48 - 8.31 (m, 4H), 8.31 - 8.14 (m, 2H), 7.36 (s, 1H), 7.28 - 7.15 (m, 2H), 5.53 - 5.44 (m, 2H), 4.70 - 4.59 (m, 1H), 4.43 (t, *J* = 5.0 Hz, 2H), 4.05 - 3.85 (m, 7H), 3.20 - 3.11 (m, 1H), 3.06 - 2.94 (m, 3H), 2.81 - 2.65 (m, 5H), 2.51 - 2.00 (m, 12H), 1.88 (br. s., 3H), 1.82 - 1.63 (m, 3H), 1.42 (s, 9H), 1.27 - 1.01 (m, 4H); HRMS (TOFMS ES+, *m/z*) exact mass $C_{53}H_{65}FN_{14}O_7S_2$ 1092.4586, found 1093.4647; LCMS (HpH Method, UV, ES) RT = 1.02 min, [M+H]$^+$ = 1093.3.

((*S*)-Methyl 2-((*S*)-2-(*tert*-butoxycarbonyl(methyl)amino)propanamido)-3,3-dimethylbutanoate (28)

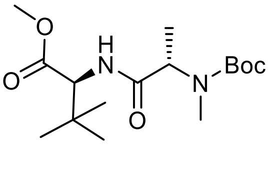

To a solution of (*S*)-methyl 2-amino-3,3-dimethylbutanoate hydrochloride (2675 g, 14.8 mol) in DCM (25 L) was added (*S*)-2-((tert-butoxycarbonyl)(methyl)amino)propanoic acid (3000 g, 14.8 mol), 4-methylmorpholine (2985 g, 29.6 mol), HOBt (2394 g, 17.7 mol) and EDC (3386 g, 17.7 mol) at 0 °C. The mixture was stirred at room temperature overnight and the mixture was then poured into 2 N $NaHCO_3$ (25 L), extracted with DCM (2 × 25L) and the combined organic layers were washed with 1M HCl (12 L) and brine, dried over Na_2SO_4, concentrated *in vacuo* to give the title product (5000 g, crude) as a glassine solid which was used for the next step without further purification.

(*S*)-2-((*S*)-2-(*tert*-Butoxycarbonyl(methyl)amino)propanamido)-3,3-dimethylbutanoic acid (29)

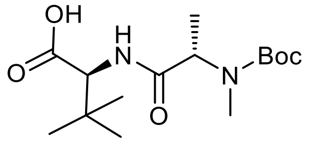

To a solution of **28** (5000 g, crude) in THF (50 L) and MeOH (25 L) was added a solution of LiOH (2545 g, 60.6 mol) in H_2O (25 L) at room temperature. The mixture was stirred at room temperature for 18 h and then treated with 1M HCl (50 L) and partitioned with EtOAc (25 L). The organic layer was washed with brine, dried over Na_2SO_4 and concentrated *in vacuo* to give the title product (4600 g, 99% yield over two steps) as a crystalline solid which was used for the next step without further purification; ^{1}H NMR ($CDCl_3$, 400 MHz): δ = 6.96 - 6.95 (m, 1H), 5.30 - 5.29 (m, 1H), 4.42 (d, *J* = 9.2 Hz, 1H), 2.79 (s, 3H), 1.48 (s, 9H), 1.32 (d, *J* = 6.8 Hz, 3H), 0.99 (s, 9H).

(2S,4R)-1-*tert*-Butyl 2-methyl 4-(tosyloxy)pyrrolidine-1,2-dicarboxylate (31)

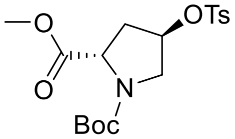

To a solution of (2S,4R)-1-tert-butyl 2-methyl 4-hydroxypyrrolidine-1,2-dicarboxylate (**30**) (4000 g, 16.3 mol) in DCM (40 L) was added DIPEA (6280 g, 48.7 mol), DMAP (141.5 g, 1.16 mol) and TosCl (3724 g, 19.6 mol) and the mixture was stirred at room temperature overnight. The reaction mixture was poured into water and separated. The organic layer was washed with 2M $NaHCO_3$, 1M HCl and brine, dried over Na_2SO_4 and concentrated *in vacuo* to give the title product (6950 g, crude) as brown oil which was used for the next step without further purification.

(2*S*,4*R*)-1-(*tert*-Butoxycarbonyl)-4-(tosyloxy)pyrrolidine-2-carboxylic acid (32)

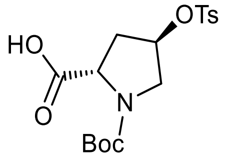

To a solution of **31,** (6950 g, 17.4 mol) in MeOH (70 L) was added a solution of LiOH (875 g, 20.8 mol) in H_2O (35 L) at 0 °C. The mixture was stirred at room temperature overnight then poured into ice water (100 L) and extracted with EtOAc (2 × 30 L). The aqueous phase was acidified to pH 3 with 1M HCl, then extracted with EtOAc (2× 50 L). The combined organic layers were washed with brine, dried over Na_2SO_4 and concentrated *in vacuo* to give the title

product (5175 g, 82% yield over two steps) as a white solid which was used for the next step without further purification; ^{1}H NMR (CDCl3, 400 MHz): δ = 7.78 (d, *J* = 8.0 Hz, 2H), 7.36 (d, *J* = 7.6 Hz, 2H), 5.02 (s, 1H), 4.44- 4.37 (m, 1H), 3.76 - 3.51 (m, 2H), 2.46 - 2.22 (m, 5 H), 1.46 - 1.39 (m, 9H).

(2*S*,4*R*)-*tert*-Butyl 2-(2,6-difluorophenylcarbamoyl)-4-(tosyloxy)pyrrolidine- 1-carboxylate (33)

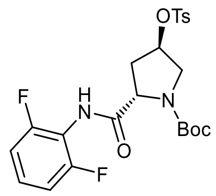

To a solution of **32** (5175 g, 13.4 mol) and 2,6-difluoroaniline (1820 g, 14.1mol) in DCM (50 L) was added DCC (2910 g, 14.1 mol), and the mixture was stirred at room temperature overnight. The mixture was filtered and the filtrate was concentrated in vacuum to give the title product (5065 g, crude) as a brown oil, which was used for the next step without purification.

(3*R*,5*S*)-5-(2,6-Difluorophenylcarbamoyl)pyrrolidin-3-yl 4-methylbenzenesulfonate (34)

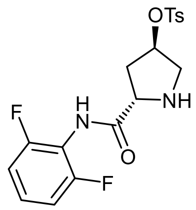

To a solution of **33** (5065 g, crude) in DCM (50 L) was added TFA (3490 g, 30.6 mol) at room temperature. The mixture was stirred at room temperature for 3 h, then concentrated *in vacuo*. The residue was diluted with water and extracted with DCM (2 × 20 L). The aqueous phase was basified to pH 8 with saturated aqueous $NaHCO_3$ and extracted with DCM (2 × 50 L). The combined organic layers were concentrated *in vacuo* to give the title product (3665 g, 66% yield) as a white solid, which was used for the next step without further purification; ^{1}H NMR ($CDCl_3$, 400 MHz): δ = 9.20 (s, 1H), 7.79 (d, *J* = 8.0 Hz, 2H), 7.36 (d, *J* = 8.0 Hz, 2H), 7.26 - 7.16 (m, 1H), 6.95 - 6.91 (m, 2H), 5.05 (t, *J* = 3.6 Hz, 1H), 4.22 (t, *J* = 8.0 Hz, 1H), 3.42 (dd, *J* = 14.0, 2.0 Hz, 1H), 2.94 (dd, *J* = 14.0, 3.5 Hz, 1H), 2.46 (s, 3H), 2.44 - 2.40 (m, 1H), 2.19 - 2.18 (m, 1H).

(3*R*,5*S*)-1-((*S*)-2-((*S*)-2-(*tert*-Butoxycarbonyl(methyl)amino)propanamido)-3,3-dimethylbutanoyl)-5-(2,6-difluorophenylcarbamoyl)pyrrolidin-3-yl 4-methylbenzenesulfonate (35)

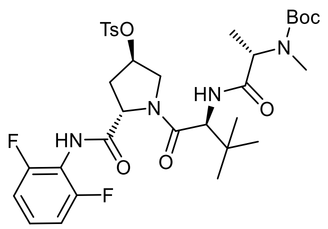

To a solution of **34** (3565 g, 9 mol) in DCM (35 L) was added **29** (3697 g, 11.7 mol), HOBt (1579.5 g, 11.7 mol) and EDC (2242.9 g, 11.7 mol) at -20 °C. The mixture was stirred at -20

°C for 1 h, then warmed to room temperature and stirred overnight. The mixture was poured into 2M $NaHCO_3$ (30 L). The organic layer was washed with 1M HCl (18 L) and brine, dried over Na_2SO_4 and concentrated *in vacuo*. The residue was purified by column chromatography (petroleum ether : ethyl acetate = 1: 1) to give the title product (3400 g, 54% yield) as a white solid; ^{1}H NMR ($CDCl_3$, 400 MHz): δ = 8.76 (s, 1H), 7.82 (d, *J* = 8.4 Hz, 2H), 7.36 (d, *J* = 8.0 Hz, 2H), 7.26 - 7.17 (m, 1H), 6.92 (t, *J* = 8.0 Hz, 2H), 5.16 (s, 1H), 4.97 - 4.94 (m, 1H), 4.73 (s, 1H), 4.52 (d, *J* = 9.2 Hz, 1H), 4.13 - 4.09 (m, 1H), 3.78 - 3.77 (m, 1H), 2.80 - 2.76 (m, 4H), 2.45 (s, 3H), 2.31 - 2.25 (m, 1H), 1.48 (s, 9H), 1.33 (d, *J* = 6.4Hz, 3H), 0.96 (s, 9H).

***tert*-Butyl (*S*)-1-((*S*)-1-((2*S*,4*S*)-4-azido-2-(2,6-difluorophenylcarbamoyl)pyrrolidin-1-yl)-3,3-dimethyl-1-oxobutan-2-ylamino)-1-oxopropan-2-yl(methyl)carbamate (36)**

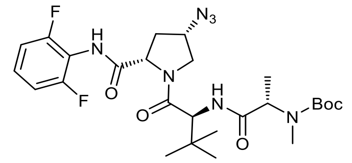

To a solution of **35** (3400 g, 4.89mol) in DMF (34 L) was added NaN_3 (413.2 g, 6.36 mol) at room temperature. The mixture was stirred at 80 °C overnight and the cooled mixture was poured into ice water (75 L) and extracted with MTBE (2 × 50 L). The combined organic layers were washed with brine, dried over Na_2SO_4 and concentrated *in vacuo* to give the title product (1200 g, crude) which was used for the next step without further purification.

***tert*-Butyl (*S*)-1-((*S*)-1-((2*S*,4*S*)-4-amino-2-(2,6-difluorophenylcarbamoyl)pyrrolidin-1-yl)-3,3-dimethyl-1-oxobutan-2-ylamino)-1-oxopropan-2-yl(methyl)carbamate (37)**

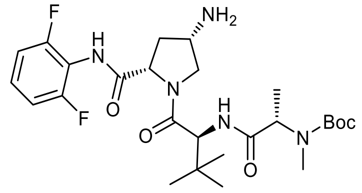

To a solution of **36** (1200 g, crude) in MeOH (12 L) was added Pd/C (120 g, l0%) at room temperature. The mixture was stirred at room temperature for 3 h under hydrogen. The mixture was filtered through a pad of celite and the filtrate was concentrated *in vacuo*. The residue was purified by column chromatography (methanol: ethyl acetate = 1: 10) to give the title product (250 g, yield) as a light yellow solid; ^{1}H NMR ($CDCl_3$, 400 MHz): δ= 9.22 (s, 1H), 7.17 - 7.15 (m, 1H), 6.94 - 6.90 (t, *J* = 8.0 Hz, 2H), 4.84 - 4.58 (m, 3H), 4.12 - 4.08 (m, 1H), 3.77 (m, 1H), 3.52 - 3.50 (m, 1H), 2.79 (s, 3H), 2.37 - 2.33(t, *J* = 6.0 Hz, 2H), 2.04 (s, 2H), 1.49 (s, 9H), 1.31 (d, *J* = 7.2 Hz, 3H), 1.01 (s, 9H); MS Calcd.:539; MS Found: 540 ([M+H$^+$]).

***tert*-Butyl 4-(3-((6-(*tert*-butylsulfonyl)-4-((4,5-dimethyl-1*H*-pyrazol-3-yl)amino)quinazolin-7-yl)oxy)propyl)piperazine-1-carboxylate (38)**

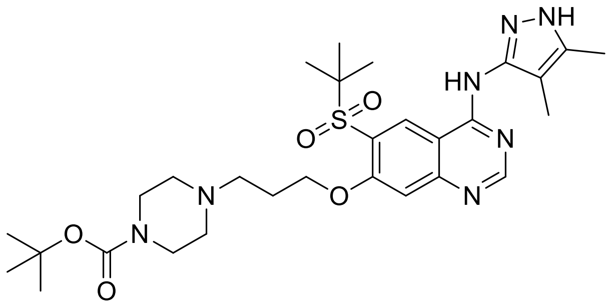

The title compound (48.0 g, 86% yield) was prepared according to similar procedure described for the preparation of **23**; ^{1}H NMR (400 MHz, DMSO-*d*$_6$) δ = 12.16 (s, 1H), 10.32 (s, 1H), 8.98 (s, 1H), 8.45 (s, 1H), 7.30 (s, 1H), 4.25 (t, *J* = 6.0 Hz, 2H), 3.43 - 3.20 (m, 4H, obscured by solvent), 2.58 - 2.51 (m, 2H), 2.40 - 2.28 (m, 4H), 2.18 (s, 3H), 1.97 - 1.87 (m, 2H), 1.74 (s, 3H), 1.39 (s, 9H), 1.32 (s, 9H); LCMS (Method A, UV, ES) RT = 0.60 min, $[M+H]^+$ = 602.3.

6-(*tert*-Butylsulfonyl)-*N*-(4,5-dimethyl-1*H*-pyrazol-3-yl)-7-(3-(piperazin-1-yl)propoxy)quinazolin-4-amine, 3 Hydrochloride (39)

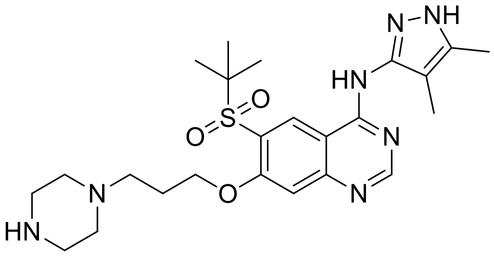

The title compound (50.27 g, 97% yield was prepared according to similar deprotection procedure described for the preparation of **24**; ^{1}H NMR (400 MHz, DMSO-*d*$_6$) δ = 12.29 (br s, 1H), 9.94 (br s, 2H), 9.23 (br s, 1H), 8.87 (s, 1H), 7.62 (s, 1H), 4.41 (t, *J* = 5.5 Hz, 2H), 3.71 (br s, 2H), 3.46 - 3.30 (m, 4H), 3.33 (4H Obscurred not observed); 2.42 - 2.27 (m, 2H), 2.21 (s, 3H), 1.80 (s, 3H), 1.35 (s, 9H); LCMS (Method A, UV, ES) RT = 0.37 min, $[M+H]^+$ = 502.3.

Methyl 5-(4-(3-((6-(*tert*-butylsulfonyl)-4-((4,5-dimethyl-1*H*-pyrazol-3-yl)amino)quinazo lin-7-yl)oxy)propyl)piperazin-1-yl)pyrazine-2-carboxylate (40)

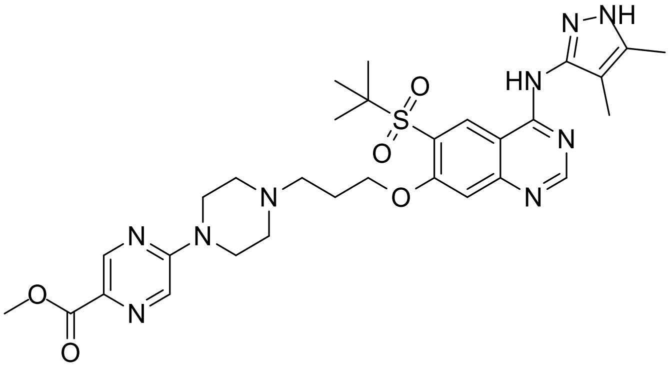

A mixture of methyl 5-chloropyrazine-2-carboxylate (0.697 g, 4.04 mmol), **39** (2.467 g, 4.04 mmol) and Et_3N (2.81 mL, 20.19 mmol) in 1,4-Dioxane (150 mL) was refluxed at 115 °C for 4 h. The reaction mixture was concentrated under reduced pressure and partitioned between water (80 mL) and DCM (100 mL). The aqueous layer was separated and extracted further with additional DCM (4 x 50 mL). The organic layers were combined, washed with saturated brine (50 mL), dried using a hydrophobic frit, and concentrated under reduced pressure. The

crude product was subjected to purification by column chromatography (340g silica column.) using an initial wash of 0-20% EtOAc in DCM over 3 column volume, followed by 5-25% MeOH in DCM over 11 column volume. The appropriate fractions were combined and the solvent was concentrated under reduced pressure to afford the title product (1.73 g, 67% yield), ^{1}H NMR (400 MHz, MeOD-d_4) δ = 8.90 (s, 1H), 8.69 (d, *J* = 1.0 Hz, 1H), 8.40 (br s, 1H), 8.21 (d, *J* = 1.0 Hz, 1H), 7.31 (s, 1H), 4.33 (t, *J* = 6.5 Hz, 2H), 3.90 (s, 3H), 3.84 - 3.74 (m, 4H), 3.35 (s, 2H), 2.73 (t, *J* = 6.5 Hz, 2H), 2.68 - 2.59 (m, 4H), 2.24 (s, 3H), 2.11 (quin, *J* = 6.5 Hz, 2H), 1.87 (s, 3H), 1.39 (s, 9H); LCMS (Method B, UV, ES) RT = 0.92 min, $[M+H]^+$ = 638.3.

5-(4-(3-((6-(*tert*-butylsulfonyl)-4-((4,5-dimethyl-1*H*-pyrazol-3-yl)amino)quinazolin-7-yl)oxy)propyl)piperazin-1-yl)pyrazine-2-carboxylic acid, *N,N*-Di-isopropylethylamine salt (41)

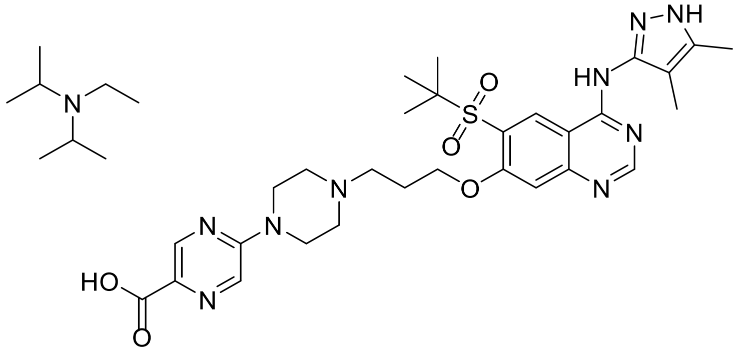

Compound **40** (20.0 g, 31.4 mmol) was suspended in methanol (140 mL), DIPEA (49.3 mL, 282 mmol) and water (50 mL). The mixture was heated at 90°C under nitrogen for 5 days. The reaction mixture was allowed to cool and the solvent was evaporated *in vacuo*. Toluene (120 mL) was added and the mixture was evaporated *in vacuo*. This was repeated twice more. To remove trace methanol the residue was dried *in vacuo* for 5 days, then dissolved in water, evaporated *in vacuo* and co-evaporated with toluene (3 x 120 mL) and dried in vacuo to give the title product (21.4 g, 91% yield) as a pale yellow solid; ^{1}H NMR (400MHz, DMSO-d_6) δ = 10.48 - 10.17 (m, 1H), 8.98 (br s., 1H), 8.64 (d, *J* = 1.2 Hz, 2H), 8.52 (br s., 2H), 8.33 (s, 2H), 8.37 - 8.32 (m, 1H), 7.32 (br s., 2H), 4.29 (t, *J* =5.9 Hz, 5H), 3.74- 3.71 (m, 6H), 3.11 – 3.04 (m, 6H), 2.18 (s, 3H), 1.99 (t, *J*= 6.5 Hz, 6H), 1.77 (s., 3H), 1.34 (s, 9H), 1.08 - 0.97 (m, 18H); LCMS (Method A, UV, ES) RT = 0.47 min, $[M+H]^+$ = 624.2.

***tert*-Butyl ((*S*)-1-(((*S*)-1-((2*S*,4*S*)-4-(5-(4-(3-((6-(*tert*-butylsulfonyl)-4-((4,5-dimethyl-1*H*-pyrazol-3-yl)amino)quinazolin-7-yl)oxy)propyl)piperazin-1-yl)pyrazine-2-carboxamido)-2-((2,6-difluorophenyl)carbamoyl)pyrrolidin-1-yl)-3,3-dimethyl-1-oxobutan-2-yl)amino)-1-oxopropan-2-yl)(methyl)carbamate (6-Boc)**

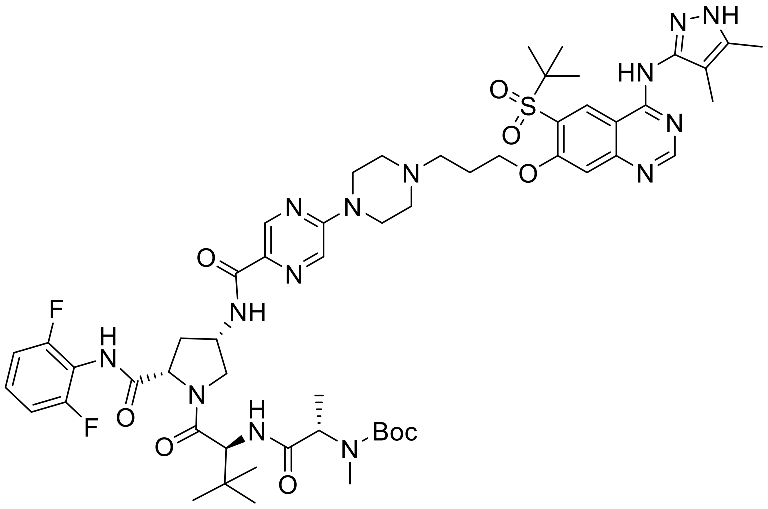

To a solution of **41** (21.4 g, 28.4 mmol) in DMSO (200 mL) at room temperature was added DIPEA (19.9 mL, 114 mmol), HOBt hydrate (8.70 g, 56.8 mmol) and **37** (14.6 g, 27.0 mmol) to give a yellow solution. To this was added EDC (10.9 g, 56.8 mmol) and the solution was stirred under nitrogen at room temperature for 20 h. The reaction mixture was poured slowly into water (600 mL) with stirring. The mixture was stirred at room temperature for 60 min and the solid was collected by filtration and washed with water (4 x 125 mL). The solid was air dried for 4 h and then dried *in vacuo* at 50°C for 18 h to give the title product (36.99 g, crude) as a pale yellow solid; ^{1}H NMR (400MHz, DMSO-d_6) δ = 9.90 (s, 1H), 8.62 (s, 1H), 8.46 (s, 1H), 8.25 (s, 1H), 7.33 (s, 4H), 7.24 - 7.11 (m, 3H), 4.65-4.60 (m, 5H), 4.48-4.46 (m, 2H), 4.29 (br s, 4H), 4.05-4.04 (m, 2H), 3.72-3.64 (m, 8H), 2.77 (br s, 4H), 2.18 (s, 4H), 1.75 (s, 5H), 1.41 (s, 9H), 1.34 (s, 9H). LCMS (Method A, UV, ES) RT = 0.82 min, $[M+2H]^{2+}$ = 573.3.

5-(4-(3-((6-(*tert*-Butylsulfonyl)-4-((4,5-dimethyl-1*H*-pyrazol-3-yl)amino)quinazolin-7-yl)oxy)propyl)piperazin-1-yl)-*N*-((3*S*,5*S*)-5-((2,6-difluorophenyl)carbamoyl)-1-((*S*)-3,3-dimethyl-2-((*S*)-2-(methylamino)propanamido)butanoyl)pyrrolidin-3-yl)pyrazine-2-carboxamide (6)

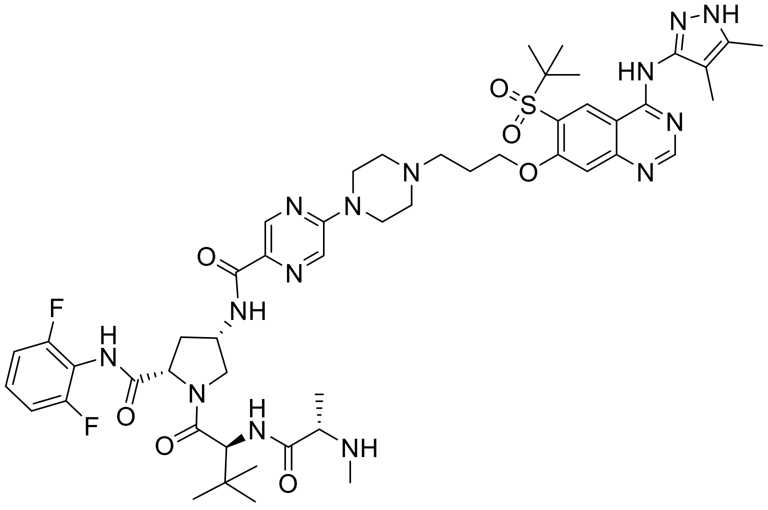

A solution of **5-Boc** (23.0g, 20.1 mmol) in DCM (40 mL) was cooled in an ice/water bath under nitrogen. To this was added TFA (46.5 mL, 603 mmol) and the mixture was stirred for 15 min at 5°C. then allowed to warm to room temperature and stirred for 1 h. The reaction mixture was evaporated *in vacuo* and the residue was dissolved in water (100 mL). To this

was added saturated aqueous sodium bicarbonate (~600 mL) to achieve pH 8. Methanol (100 mL) was added and the mixture was stirred rapidly for 3 h. The resulting solid was collected by filtration, washed with water and air-dried to give the title product (15.93 g, 76% yield) as a pale yellow solid; ^{1}H NMR (600 MHz, DMSO-d_6) δ = 12.46 - 11.85 (m, 1H), 10.32(br s, 1H), 9.90 (s, 1H), 8.98 (br s, 1H), 8.75 (d, *J* = 8.3 Hz, 1H), 8.61 (d, *J* = 1.3 Hz, 1H), 8.48 – 8.42 (m, 1H), 8.25 (s, 1H), 7.84 (d, *J* = 9.2 Hz, 1H); 7.36 - 7.32 (m, 1H), 7.33 - 7.30 (m, 1H), 7.14 (t, *J* = 8.1 Hz, 2H), 4.67-4.58 (m, 2H), 4.47 (d, *J* = 9.3 Hz, 1H); 4.28 (br t, *J* = 5.8 Hz, 2H), 4.07 (dd, *J* = 10.2, 6.6 Hz, 1H), 3.74-3.67 (m, 4H), 3.64 (dd, *J* = 10.2, 6.5 Hz, 1H), 2.98 (q, *J* = 6.9 Hz, 1H),), 2.64-4.54 (m, 3H), 2.53-2.51 (m, 4H), 2.20 (s, 3H), 2.17 (s, 3H), 2.09-2.00 (m, 1H), 2.00-1.94 (m, 2H), 1.75 (br s, 3H), 1.33 (s, 9H), 1.13 (d, *J* = 6.9 Hz, 3H), 0.93 (s, 9H); ^{13}C NMR (DMSO-d6,151MHz: δ =174.0(s,1C),170.5 (s, 1C),169.4 (s,1C), 163.5 (s,1C), 159.5 (br s, 2C), 157.9 (br s,1C), 157.6 (dd, *J*=249.5, 5.4 Hz, 2C), 155.0 (s,1C), 154.7 (br s,1C), 141.6 (s, 1C), 132.4 (s, 1C), 132.2 (br s, 1C), 128.8 (s,1C), 127.8 (br t, *J*=9.7 Hz, 1C), 123.6 (s, 1C), 114.3 (t, *J*=16.9 Hz, 1C), 111.5 - 111.9 (m, 2C), 108.8 (br s, 1C), 108.0 (s, 1C), 107.3 (br s, 1C), 67.1 (s, 1C), 60.6 (s, 1C), 59.2 (s, 1C), 58.3 (s, 1C), 55.9 (s, 1C), 53.9 (s, 1C), 53.0 (s, 1C), 52.3 (s, 2C), 48.0 (s, 1C), 43.9 (s, 2C), 35.1 (s,1C), 34.3 (s, 1C), 34.3 (s, 1C), 26.1 (s, 3C), 25.7 (s, 1C), 23.4 (s, 3C), 19.0 (s, 1C), 9.6 (br s, 1C), 7.8 (br s, 1C); HRMS (TOFMS ES+, m/z) exact mass $C50H66F_2N14O7S$ 1044.4928, found 889.2910; LCMS (Method B, UV, ES) RT= 1.01 min, $[M+2H]^{2+}$ = 523.4.

References

1 Schwanhausser, B. *et al.* Global quantification of mammalian gene expression control. *Nature* **473**, 337-342, doi:10.1038/nature10098 (2011).

2 Mathieson, T. *et al.* Systematic analysis of protein turnover in primary cells. *Nat Commun* **9**, 689, doi:10.1038/s41467-018-03106-1 (2018).
