# Supplementary material for: Extended pharmacodynamic responses observed upon PROTAC-mediated degradation of RIPK2
Source: Commun Biol. 2020 Mar 20;3:140. doi: 10.1038/s42003-020-0868-6 (PMC7083851; doi:10.1038/s42003-020-0868-6)
Supplement: Supplementary file 6 — Reporting Summary [file 42003_2020_868_MOESM6_ESM.pdf]

nature research

Corresponding author(s): John D Harling

Last updated by author(s): Feb 20, 2020

Reporting Summary

Nature Research wishes to improve the reproducibility of the work that we publish. This form provides structure for consistency and transparency in reporting. For further information on Nature Research policies, see Authors & Referees and the Editorial Policy Checklist.

Statistics

For all statistical analyses, confirm that the following items are present in the figure legend, table legend, main text, or Methods section.

n/a	Confirmed	
☐	☒	The exact sample size (*n*) for each experimental group/condition, given as a discrete number and unit of measurement
☐	☒	A statement on whether measurements were taken from distinct samples or whether the same sample was measured repeatedly
☐	☒	The statistical test(s) used AND whether they are one- or two-sided<br>*Only common tests should be described solely by name; describe more complex techniques in the Methods section.*
☐	☒	A description of all covariates tested
☐	☒	A description of any assumptions or corrections, such as tests of normality and adjustment for multiple comparisons
☐	☒	A full description of the statistical parameters including central tendency (e.g. means) or other basic estimates (e.g. regression coefficient) AND variation (e.g. standard deviation) or associated estimates of uncertainty (e.g. confidence intervals)
☐	☒	For null hypothesis testing, the test statistic (e.g. *F*, *t*, *r*) with confidence intervals, effect sizes, degrees of freedom and *P* value noted<br>*Give P values as exact values whenever suitable.*
☒	☐	For Bayesian analysis, information on the choice of priors and Markov chain Monte Carlo settings
☒	☐	For hierarchical and complex designs, identification of the appropriate level for tests and full reporting of outcomes
☒	☐	Estimates of effect sizes (e.g. Cohen's *d*, Pearson's *r*), indicating how they were calculated

Our web collection on statistics for biologists contains articles on many of the points above.

Software and code

Policy information about availability of computer code

Data collection	Odyssey Scanner CLX, Sally Sue Simple Western, MSD Sector imager 6000,
Data analysis	Image Studio Lite 5.2, Compass Protein Simple 3.1.7, MSD DISCOVERY WORKBENCH 4.0.12.1, GraphPad Prism 5.0.4

For manuscripts utilizing custom algorithms or software that are central to the research but not yet described in published literature, software must be made available to editors/reviewers. We strongly encourage code deposition in a community repository (e.g. GitHub). See the Nature Research guidelines for submitting code & software for further information.

Data

Policy information about availability of data

All manuscripts must include a data availability statement. This statement should provide the following information, where applicable:

- Accession codes, unique identifiers, or web links for publicly available datasets
- A list of figures that have associated raw data
- A description of any restrictions on data availability

The source data underlying Figure 1 is in the Supplementary Information file. The source data underlying Figures 2-6 is available on reasonable request from the corresponding author. The source data for the proteomics plots in Figure 7 are included in the Supplementary spreadsheets TPP_supplement, mPDP_supplement and AEC_supplement. Correspondence and requests for data materials should be addressed to JDH (john.d.harling@gsk.com).

Field-specific reporting

Please select the one below that is the best fit for your research. If you are not sure, read the appropriate sections before making your selection.

☒ Life sciences ☐ Behavioural & social sciences ☐ Ecological, evolutionary & environmental sciences

For a reference copy of the document with all sections, see nature.com/documents/nr-reporting-summary-flat.pdf

Life sciences study design

All studies must disclose on these points even when the disclosure is negative.

Sample size	Number of animals per group was selected based on power analysis calculations that estimated an 80% power to detect a reduction of 80% or greater in TNFa and 50% or more for RIPK2 using sample sizes of at least 5 per group.
Data exclusions	For samples analysed using Protein Simple capillary system the exclusion criterion was a signal to noise ratio below 10 for the housekeeping protein (No data were excluded). For classical immunoblotting the exclusion criterion was 50% or more variation of the housekeeping protein levels. Samples for RIPK2 analysis (120h) fig 5a were excluded based on this criterion. For cytokines the exclusion criteria were: less than 5-fold increase compared to unstimulated samples (for DMSO/vehicle samples) and more than 10-fold difference between replicates. No data were excluded
Replication	Some doses were re-included in follow-up studies to verify the reproducibility of data (see figure 3b)
Randomization	A randomization application was used ,allocating animals to treatment groups via animal number for all studies except for the in vivo L18-MDP stimulation study where randomization was carried out based on bodyweight.
Blinding	Blinding was conducted for the in vivo L18-MDP stimulation study only.

Reporting for specific materials, systems and methods

We require information from authors about some types of materials, experimental systems and methods used in many studies. Here, indicate whether each material, system or method listed is relevant to your study. If you are not sure if a list item applies to your research, read the appropriate section before selecting a response.

Materials & experimental systems

n/a	Involved in the study
☐	☒ Antibodies
☐	☒ Eukaryotic cell lines
☒	☐ Palaeontology
☐	☒ Animals and other organisms
☐	☒ Human research participants
☒	☐ Clinical data

Methods

n/a	Involved in the study
☒	☐ ChIP-seq
☒	☐ Flow cytometry
☒	☐ MRI-based neuroimaging

Antibodies

Antibodies used	Rabbit anti-RIPK2 (Cell Signaling, 4142, lot 1 from 09/2016 – classical immunoblotting and lot 1 from 03/2017 and lot 1 from 08/2018 – capillary immunoblotting), mouse anti-β tubulin (Sigma, T8328, lot045M4763V), mouse anti-β-actin (Abcam, ab6276, lot GR231981-4), mouse anti-actin (Sigma, A2228), rabbit anti-cIAP1 (Cell Signaling, 7065, lot 1 from 12/2016), rabbit anti-cIAP2 (Cell Signaling, 3130, lot 6 from 12/2016), mouse anti-XIAP(BD Biosciences, 610717, lot 4171976), rabbit anti-β-actin (Abcam, ab8227, lot GR3188015-2), mouse anti-vinculin (Abcam, ab129002, GR221671-55), donkey anti-rabbit IRdye 800CW (Licor, 926-32213, lot C60119-11), donkey anti-mouse IRdye 680RD (Licor, 926-68072, lot C60217-15), secondary rabbit antibody-HRP 1x concentrated (ProteinSimple, 042-206, lot #83875), Secondary Rabbit Antibody-HRP 20x Protein Simple, 043-426, lot #82621), Secondary Mouse Antibody-HRP (Protein Simple 042-205, lot #81675), Secondary Rabbit Antibody-HRP (ProteinSimple, 042-206, lot #88566).
Validation	Antibodies were used for classical immunoblotting at the concentrations recommended by the manufacturer. For capillary immunoblotting a step of protein concentration versus antibody dilution was carried out and the combination of the two where the antibody saturation was achieved without burn-out of the signal was selected.

Eukaryotic cell lines

Policy information about cell lines

Cell line source(s)	ATCC

Authentication | None of the cell lines were authenticated beyond that conducted by the supplier

Mycoplasma contamination | All cell lines were negative for mycoplasma

Commonly misidentified lines (See ICLAC register) | *Name any commonly misidentified cell lines used in the study and provide a rationale for their use.*

Animals and other organisms

Policy information about studies involving animals; ARRIVE guidelines recommended for reporting animal research

Laboratory animals | male Sprague Dawley rats(7-10 weeks old on dosing), male CD or Wistar Han rats (7 - 9 weeks old on dosing), female CD rats (7-8 weeks at arrival)

Wild animals | *Provide details on animals observed in or captured in the field; report species, sex and age where possible. Describe how animals were caught and transported and what happened to captive animals after the study (if killed, explain why and describe method; if released, say where and when) OR state that the study did not involve wild animals.*

Field-collected samples | *For laboratory work with field-collected samples, describe all relevant parameters such as housing, maintenance, temperature, photoperiod and end-of-experiment protocol OR state that the study did not involve samples collected from the field.*

Ethics oversight | GlaxoSmithKline

Note that full information on the approval of the study protocol must also be provided in the manuscript.

Human research participants

Policy information about studies involving human research participants

Population characteristics | Crohn's Disease (CD) or Ulcerative Colitis (UC) patients at St Bartholomew's Hospital, London

Recruitment | N/A as not clinical study

Ethics oversight | IRB/EC approved protocol

Note that full information on the approval of the study protocol must also be provided in the manuscript.
