# Supplementary material for: Extended pharmacodynamic responses observed upon PROTAC-mediated degradation of RIPK2
Source: Commun Biol. 2020 Mar 20;3:140. doi: 10.1038/s42003-020-0868-6 (PMC7083851; doi:10.1038/s42003-020-0868-6)
Supplement: Supplementary file 8 — Description of Supplementary Files [file 42003_2020_868_MOESM8_ESM.docx]

**Description of Additional Supplementary**

**Files File Name: Chemdraw Figure 1**

**Description:** Structures used in Figure 1

**Files File Name: Chemdraw Figure 2**

**Description:** Structures used in Figure 2

**Files File Name: Supplementary Data 1**

**Description:** Data used in Figures 1-6

**File Name: Supplementary Data 2**

**Description:** Affinity Enrichment Chemoproteomic and Kinobead data

**File Name: Supplementary Data 3**

**Description:** Thermal Proteome Profiling data

**File Name: Supplementary Data 4**

**Description:** Multiplexed proteome dynamics profiling data
